
JOURNAL INFORMATION
==============================
NLM Title Abbreviation: J Orthop Traumatol
Journal ID: J Orthop Traumatol
NLM Journal ID: 101090931

Journal of Orthopaedics and Traumatology : Official Journal of the Italian Society of Orthopaedics and Traumatology

ISSN: 1590-9921
EISSN: 1590-9999
Publisher: Springer

ARTICLE INFORMATION
==============================
PMCID: PMC4417754
DOI: 10.1007/s10195-012-0208-9
Article ID: 208
Article version: 1
Subjects: Events

Oral Communications

Electronic publication date: 2012 Aug 30
Print publication date: 2012 Nov
Volume: 13
Issue: Suppl 1
First page: 1
Last page: 24
Copyright: © The Author(s) 2012
License: Open AccessThis article is distributed under the terms of the Creative Commons Attribution 2.0 International License (https://creativecommons.org/licenses/by/2.0), which permits unrestricted use, distribution, and reproduction in any medium, provided the original work is properly cited.
License URL: https://creativecommons.org/licenses/by/2.0/

Keywords: Anterior Cruciate Ligament, Anterior Cruciate Ligament Reconstruction, Radial Head, Unicompartmental Knee Arthroplasty, Constant Score
issue-copyright-statement: © The Author(s) 2012

==============================
J Orthopaed Traumatol (2012) 13 (Suppl 1):S1–S24
DOI 10.1007/s10195-012-0208-9

10 NOVEMBER 2012

### ORAL COMMUNICATIONS

### C01—HIP 1

### Utility of intraoperative navigation to reduce the incidence of limb length discrepancy after total hip arthroplasty. A prospective comparative study

L. Orlandini[1], V. Meroni[1], F. Carlea[1], V. Sansone[2], M. Ulivi*[1]

[1]Unità Operativa di Ortopedia e Traumatologia, Istituti Clinici Zucchi (Monza, IT);
[2]Clinica Ortopedica dell'Università degli Studi di Milano, Istituto Ortopedico Galeazzi IRCCS (Milan, IT)

**Introduction** Limb length discrepancy after total hip replacement is a major cause of discomfort and dissatisfaction for patients and may also be the cause of abnormal stress transmission through the joint therefore contributing to mobilization and early failure of the implant. Limb length discrepancy correction is also linked to best offset reproduction, fundamental for the long term stability of the implant. Study objective: validation of intraoperative limb length and femoral offset measure accuracy with BrainLab navigation, when compared to manufacturing specifications of trial femoral neck (standard or high-offset) and ball head length.
**Materials and methods** Sixty consecutive patients (11/2010–11/2011) with primary or secondary coxarthritis underwent total hip arthroplasty. 12 male, 48 female. Mean age: 67.83 (37–84) Mean BMI: 26.26. Trilock stem, Pinnacle cup, Marathon polyethylene liner (De Puy) Biolox ceramic head. Navigation: BrainLab, Express Hip 5.1 Software. With definitive trial rasp "in situ" navigation assisted limb length measurements have been conducted, utilizing the different available head/neck combinations (short, medium, long/with, without offset) Each patient underwent limb length and femoral offset intraoperative measurement.
**Results** Intraoperative measurements with BrainLab navigation have been reported in differences expressed in millimeters among the different trial components when compared to the respective manufacturing specifications. Results show a mean difference of −0.17 mm e 0.14 mm for offset and limb length measurement respectively (SD ± 1.24 mm), among nominal values of trial components and those recorded with navigation.
**Discussion** Limb length and offset intra-operative measurement with the aid of computer assisted BrainLab, Software Express Hip 5.1 navigation, proved to be a valid, accurate and immediate instrument to match intraoperatively in an objective manner the differences in measurements while changing the trial components (stem neck and ball heads).
**Conclusions** The computer assisted BrainLab, Software Express Hip 5.1 navigation, is a valid, precise and reproducible instrument for the intra-operative limb length and offset determination.

### Complications in hip viscosupplementation

P. Piciocco*[1], F. Cavatorta[2], N. Santori[3], F.S. Santori[3]

[1]Policlinico Umberto I (Rome, IT);
[2]Ospedale Civile di Trento (Trento, IT);
[3]Rome American Hospital (Rome, IT)

**Introduction** The viscosupplementation (VS) with hyaluronic acid reduces pain and consequently consumption of non-steroidal drugs that are responsible for an increase in morbidity and mortality due to gastrointestinal and cardiovascular diseases. Furthermore the VS allows to delay the intervention surgery. There are several types of aids used, depending on the different molecular weight. Several methods are used to increase the precision: fluoroscopic or ultrasound guidance. The VS has a low risk of complications; the aim of this study is to describe them.
**Materials and methods** Our experience is based on 2,178 cases in 617 patients classified as grade II–IV according to Kellgren and Lawrence classification. In 161 patients was practiced VS by ultrasound guided injections ones a week for three times, group A. In 456 patients was used a stabilized hyaluronic acid of non animal origin (NASHA) with fluoroscopic guidance. The administration was performed every 6 months for three times (group B). The results were verified by VAS, Walking pain and Lesquerne index. We report 2 cases that came to our attention with septic arthritis after VS.
**Results** The decrease in VAS, Walking Pain and Lesquerne index was statistically significant ($p < 0.001$) in both groups. A slight local discomfort was observed in 4 % of patients in group B with remission within 7 days. There was 1 case of severe anemia with retroperitoneal hematoma and 1 case of septic arthritis regressed after antibiotic therapy. In the other 2 cases of septic arthritis an arthroscopic debridement and antibiotic therapy were performed with a complete resolution of infection.
**Discussion** The VS is universally considered to be without complications. The increasing in pain with the use of NASHA is described and is present only in the group (B) of our series. The retroperitoneal hematoma and the cases of septic arthritis, including those received under our observation, were observed in group A (but we have to emphasize that in group B we used antibiotics for 2 days after infiltration).
**Conclusions** The viscosupplementation of the hip is, like the ones in the knee, a viable therapeutic option in the treatment of osteoarthritis especially in the young patients. It is important to keep in mind the possible complications for a right information of the patient.

### Comparative analysis of pain in stemless hip implants

G. Logroscino*, E. Pagano, A. Ziranu, G. Malerba, V. Ciriello, C. Giusti, C. Fabbriciani

Dipartimento di Scienze Ortopediche e Traumatologiche, Università Cattolica-Policlinico A. Gemelli (Rome, IT)

**Introduction** Stemless hip implants have been proposed assuming the advantage of "tissue sparing surgery", and of bone-stock preservation in case of revision. The new stems design, allowing a more "physiologic" load transmission to the bone and a less soft and bone-tissue injury, seems to confer a better control of post-operative pain. To evaluate pain and functional outcomes in two homogeneous populations of primary THA in which stemless prosthesis and traditional long stems were implanted.
**Materials and methods** Thirty patients underwent to THA surgery and were divided into two groups of 15 patients: in the first group, a neck preserving stemless prosthesis (Proxima-DePuy) and, in the second group, a straight-anatomical stem (Stryker-Symax) were implanted. All the patients were evaluated at 1, 3, 6 and 12 months by radiographic examination and clinical assessment (HHS, WOMAC, VAS).
**Results** The groups were homogeneous for age (I: 69; II: 63 years) and sex. The prosthetic components resulted bony stable osseointegrated in all cases. The mean follow-up was 12 months. There were no major complications. At 6 months both groups showed no differences at HHS score and WOMAC, whereas there was a significant difference in the rate of residual pain (VAS Group I: 18.3 mm, and VAS Group II: 38 mm). At 1 year follow-up both groups showed a good functional outcome and pain control (HHS-I: 94.4; II: 92.9; WOMAC-I: 7 %; II: 11.2 %; VAS-I: 4 mm, II: 4.8 mm) without significant differences between the groups.
**Discussion** Both implants provided an adequate restoration of joint function in the absence of pain at 1 year. At 6 months, patients treated with stemless prostheses demonstrated better control of pain, probably correlated to less invasive surgery and to the physiological load transmission.
**Conclusions** The stemless THA allows the use of less invasive surgical approach and a greater saving of the bone stock. This study demonstrated that these implants can provide a better control of pain in the early postoperative period, and earlier return to daily life activity.

### Pulsed electromagnetic field (PEMF) stimulation in patients undergoing revision of hip arthroplasty. Randomized double blind prospective study

D. Dallari*[1], N. Del Piccolo[1], M. Filanti[1], N. Rani[1], C. Carubbi[1], C. Stagni[1], M. Fini[2]

[1]S.S.D. Chirurgia Conservativa e Tecniche Innovative, Istituto Ortopedico Rizzoli (Bologna, IT);
[2]S.S.D. Laboratorio Studi Preclinici e Chirurgici, Istituto Ortopedico Rizzoli (Bologna, IT)

**Introduction** Poor quality of bone-stock can cause poor results in revision surgery of hip. Strategies to improve the periprosthetic bone formation are useful supplement in surgical treatment of prosthetic loosening. Pulsed Electromagnetic Fields are a local stimulation, safe, non invasive, with results proved in numerous clinical and preclinical studies. We present our results in a prospective, randomized, double-blind trial.
**Materials and methods** Thirty patients were subjected to revision of hip arthroplasty. Revisions were performed using Wagner SL revision stem with a trans-femoral approach. The clinical evaluation was performed with Merle-D'Aubigné classification. We conducted a densitometric evaluation of periprosthetic femoral bone post-operative and at 90 day of follow-up. Change of more than 3.5 % was considered as positive response. One half of the treated cases were subjected to PEMF stimulation, which began in the seventh postoperative day and continued until the nineteenth day. Patients used a PEMF generator (Biostim, Igea, Carpi—Modena, Italy) for at least 6 h a day.
**Results** At 90 days the patients treated with PEMF had a statistically significant ($p < 0.05$) increase in functionality according to the classification Merle D'Aubigné. In general, the increase in bone mineral density (BMD) was most evident in patients treated with PEMF, even if not statistically significant. In Gruen zones 5 and 6, corresponding to the medial femoral cortex, we observed a 40 % positive responses in both areas in the control cases and respectively 93 and 66 % in cases with to stimulation ($p < 0.05$).
**Discussion** In this study we examined the effect of PEMF stimulation in patients undergoing revision hip arthroplasty. Densitometric evaluation observed a greater number of cases responsive in Gruen zones 5 and 6. We also found a significantly greater clinical improvement in patients treated with PEMF compared to control cases. There was no negative effect.
**Conclusions** Our study shows that PEMF stimulation has a positive impact on the recovery of periprosthetic bone tissue and on functional recovery after surgery. We think that PEMF cloud be a useful integration in the surgical treatment of prosthetic loosening, especially in cases with severe bone loss and in the elderly.

### *C02—TRAUMATOLOGY 1*

### Intra-articular impacted fragments in acetabular fractures

G. Rocca*, M. Spina

USO di Traumatologia, Azienda Ospedaliera Universitaria Integrata di Verona (Verona, IT)

**Introduction** The fractures of the posterior wall represent about 50 % of all acetabular fractures if we consider both the isolated fractures and those associated with other types of bone lesions. These fractures are frequently associated with a dislocation or subluxation of the femoral head. 35–50 % of the dislocations, once reduced, are complicated with intra-articular fragments, which must be surgically removed to prevent serious damage of the cartilage and a painful arthritis. Impacted fragments, after a Letournel type II posterior wall fracture, change the sphericity of the acetabular cavity and can cause joint instability and hip impingement. These fragments must be replaced. The anatomy of the acetabulum has to be rebuilt, often using bone grafts. Otherwise cartilage consumption, degenerative phenomenons, femoral head and acetabular necrosis are most common.
**Materials and methods** Through many intra-operative images we show step-by-step the impacted fragments relocation, their relocation using also bone grafts or its synthetic substitutes. We show also the most effective technique in removing intra-articular fragments.
**Results** During 10 years we treated 118 posterior wall fractures associated with isolated and complicated lesions. In 38 cases intra-articular fragments were removed; in 19 cases the fragments were reduced. In 18 patients hip pain increased with the load 6 months after surgery. In 8 cases necrosis of the femoral head appeared (15–30 months after surgery). In 11 cases was carried out total hip arthroplasty.
**Discussion** In 35–50 % of cases, intra-articular bone fragments are impacted in the acetabulum after femoral head reduction. Dislocation is a common complication of a posterior wall fracture. It's easy understand that poor outcomes are greatly reduced in cases surgically treated (15–30 %) than in cases conservatively treated (88 %).
**Conclusions** The removal of the fragments from the acetabular cavity is therefore a mandatory technique for the functional hip restoration, otherwise destinated to a painful crippling arthritis. After every pure hip dislocation a CT scan must be performed to check the presence of possible fragments. Kocher-Langebeck is the most common approach used for intra-articular impacted fragments removal.

### Treatment of nonunion aseptic femur either with intramedullary nail or with plate and graft

M. Di Liddo*, L. Cevolani, M. Baldassarri, T. Frisoni, D.M. Donati, S. Giannini

Istituto Ortopedico Rizzoli (Bologna, IT)

**Introduction** When a fracture has no independent capacity to heal from the trauma to 9 months and shows no signs of clinical and radiographic consolidation, it is called pseudoarthrosis. The pseudarthrosis represents a failure of the system of surgical reduction and fixation of a fracture that leads to the failure evolution of reparative bone callus.
**Materials and methods** In the last 10 years, at the Orthopedic Clinic of the Institute Rizzoli in Bologna, 18 patients with nonunion aseptic femur were treated. 8 cases were treated with the intramedullary nail fixation and 10 cases with the graft and plate fixation with cortical omoplastic opposed. The 8 cases of pseudoarthrosis treated with nail derived from all closed fractures initially treated with intramedullary nail (7 cases) and with plate (1 case). The synthesis of pseudoarthrosis was performed in 6 cases with nail and blocked in 2 cases with nail dynamic. The 10 cases of pseudoarthrosis treated with the technique of plaque and graft versus cortical derived from fractures closed (9 cases) and in 1 case with exposure punctiform treated initially with external fixator. The other 9 patients were initially treated with intramedullary nail (3 cases) and with a straight plate (7 cases).
**Results** Among the 8 nonunion treated with intramedullary nail there was consolidation in two cases, while in 6 cases, the consolidation has occurred on average after 6 months. Among the 10 nonunions treated with the technique of cortical plate and graft, consolidation occurred on average after 6 months; in 3 cases a new revision of the cortical plate and graft was performed.
**Conclusions** The intramedullary nail is an excellent method of synthesis for the treatment of nonunions of the femur in particular because it allowed us to avoid a large detachment of the periosteum and devascularization of the stumps of nonunion with a relatively simple surgical technique, however, must be absolutely avoided, because the static clamping prevents proper contact between the stumps. The treatment of pseudoarthrosis with plaque and graft versus cortical has the disadvantage of having to detach the widely outbreak of pseudoarthrosis and being a more complex surgical technique is fraught with major complications, but the results are equally valid.

### Treatment of diaphyseal fractures of the tibia by intramedullary locked nailing

E. Marchese, S. Cannavò*, D. Garghozloo, A. Raffa, G. Bruno

(Catania, IT)

**Introduction** Intramedullary locked nailing currently represents the treatment of choice in most cases of diaphyseal fractures of the tibia. Indeed non-operative treatment today is recommended less often as it requires long hospitalization times, and delay in the granting of the load, due to frequent faults consolidation. The excellent stability obtained by nail allows early and active mobilization of the limb granting, in some cases, immediate and full load. The flexibility of fixation system provides significant advantages in terms of callus formation.
**Materials and methods** At the Orthopaedic Clinic of the University of Catania, in the period between January 2008 and December 2011, 158 patients were treated by intramedullary nailing technique, of which 117 were males and remaining females with average age of 44 years (range 18–72 years). Fractures were mostly closed or with punctiform exposure and were classified by AO classification. Patients were evaluated clinically and radiographically at 1, 3, 6 and 12-month follow-up. In some cases dynamization of the intramedullary nail was performed.
**Results** Results at follow-up revealed fracture healing without complications in 87 % of cases, we found in the remaining cases delays in consolidation, pseudoarthrosis and malunion, but no case of infection.
**Discussion** Our experience with intramedullary nail of the tibia was positive, with short times of consolidation and an early grant of the load. The results are satisfactory: this fact encourages us therefore continue this way. The anaesthetic and surgical risk, currently very reduced, do not justify the choice of a conservative treatment that requires a long hospital stay, a poor quality of life during treatment not protecting the patient from possible defects of consolidation.
**Conclusions** According to our experience, the treatment based on reduction and fixation with intramedullary nail can be considered the first choice of treatment in case of closed fractures or with first-type exposure (Gustilo-Anderson rating scale). Moreover it ensures a rapid functional recovery of limb and is well tolerated by patients.

### External fixation for the treatment of calcaneal fractures: focus on load and follow-up

D. Speciale*, V. Caiaffa, M. Sperti

Asl Taranto, Ospedale "SS Annunziata" (Taranto, IT)

**Introduction** The treatment of calcaneal fractures is controversial. No treatment has been shown to be superior to others. The open reduction with internal fixation through the lateral approach has been to date the gold-standard, for the chance to reduce the articular fragments.
**Materials and methods** For some time the minimally invasive techniques have become remarkably popular, especially when you have to treat patients with severe soft tissue and local or systemic contraindications. In particular, we used a dedicated mini fixator, with 6 chips, which realizes a distraction to three points in the longitudinal, angular and cranio-caudal direction. The surgical technique provides closed and minimal invasive reduction, under scopic control. Then one proceeds to the positioning of Kirschner, chips and external fixator, with distraction, if necessary. By November 2009 and December 2011 we treated 27 fractures of the calcaneus in 24 patients (three bilateral) with a mean age of 46 aa (min 29 max 84). The surgery was performed on average after 5 days.
**Results** Our absolute indication was given for polytraumatized patients, suffering skin, open wound; surgery was planned in the remaining cases. The surgical outcome was satisfactory in all cases, while keeping the chip in some cases was not optimal (correct position). We gave the load to 10–11 weeks and the fixator has been removed at 12 weeks, until March 2011. In the next 9 treated cases, we allowed the partial (50 %) load at 5 weeks and complete load at 8 weeks. All cases, however, healed in 12 weeks. We evaluated the outcome using the scoring system Maryland Foot Score, with an average of 79/100. It is interesting to note that those patients who have advanced the partial load scored comparable to the others, with Maryland Foot Score average 80/100. We had no cases of deep infection, only a few cases of superficial infection. In one case we performed subtalar arthrodesis for persistent pain. One case was lost during follow-up.
**Discussion** The main goal of treatment of displaced fractures of the calcaneus should be the restoration of three-dimensional structure.
**Conclusions** The early load will be elucidated in larger series.

### *C03—SHOULDER AND ELBOW 1*

### Clinical outcome of arthroscopic repair of massive cuff tears: a comparative study of immobile versus mobile

P. Randelli, P.A. Arrigoni, L. Zottarelli *, C. Fossati, P. Spennacchio, P. Cabitza

Policlinico San Donato, Università degli Studi di Milano (San Donato Milanese, Milan, IT)

**Introduction** The purpose of this study was to compare clinical outcomes of retracted massive cuff tears treated using an interval slide releases technique if immobile versus cuff repair without interval slide if mobile.

**Materials and methods** 25 patients that underwent arthroscopic repair for massive rotator cuff tears were divided in two groups. In group 1, a single or double interval slide release was performed to achieve an adequate tendon mobilization. In group 2 massive cuff tears were arthroscopically repaired without this additional release. Patients were retrospectively evaluated with validated outcomes scores: Constant Score (CS), pain score (VAS) and Single Assessment Numeric Evaluation (SANE).

**Results** The two groups were comparable for age (group 1: 63 ± 6; group 2: 69 ± 7) gender (% male, group 1: 61 %; group 2: 50 %) and operated dominant side (group 1: 61.5 %; group 2: 75 %). The mean follow-up for group 1 and 2 were 31 and 28 months respectively ($p = 0.4$). The two groups showed no significant difference in SANE and VAS evaluation (group 1: SANE 77 %, VAS 1.3; group 2: SANE 88 %, VAS 1.6). No significant difference was found between the two groups for the CS (group 1: 66.5 ± 11; group 2: 75 ± 14; $p = 0.1$). Subjective CS for group 1 and 2 were 31 ± 5 and 30.8 ± 7 respectively ($p = 0.9$). A statistical significant difference was found for objective CS in the control group (group 1: 35.5 7; group 2: 44 ± 8; $p = 0.009$).

**Conclusions** Subjectively clinical outcomes of arthroscopic repair in rotator massive cuff tears, immobile or mobile are comparable and satisfactory. Although objectively immobile cuff tears show inferior results despite interval slide technique.

### Quality of life and cost-effectiveness analysis in the surgical treatment of elbow stiffness

G. Giannicola, G. Bullitta*, F. Sacchetti, M. Scacchi, G. Citoni

“Sapienza” Università di Roma (Rome, IT)

**Introduction** The purposes of this study were to examine the quality of life (QF) improvement achieved after open surgical treatment of elbow stiffness and to verify the cost/effectiveness ratio (CER) of these surgical procedures.

**Materials and methods** Between 2007 and 2010, 33 patients (22 males, 11 females; mean age 49 years) underwent surgical treatment for elbow stiffness. Stiffness was caused by osteoarthritis in 5 patients, distal humeral nonunion in 2, trauma in 24, and rheumatoid arthritis in 2. 14 Humeral-ulnar arthroplasty (HUA), 6 HUA associated to radiocapitellar replacement, 5 HUA associated to radial head replacement and 8 total elbow arthroplasty were performed. All patients were evaluated pre-and post-operatively with MEPS, M-ASES and DASH scores. We used SF-36 to assess QF. The CER was evaluated with QALYs. Correlation between some explanatory variables (range of movement, pain, age, sex, dominant arm involvement, operated on side, aetiology, type of surgery, previous surgeries and the presence of ulnar neuropathy, elbow scores) and QF improvement was assessed.

**Results** The mean follow-up was 26 months (range 12–48). The average increase of MEPS and m-ASES score was 43 (49–92) and 41 (44–85), respectively. The average decrease in DASH and m-ASES pain score was 44 (59–15) and 21 (21–43), respectively. The improvement of SF-36 Physical Component Summary score and the SF-36 Mental Component Summary score was 7.6 and 7, respectively. The improvement in QF, expressed in QALYs value, was on average about 6,000 Euros per capita per year. All surgical procedures have shown a good CER with a value between 670 and 817 Euros per QALY. DASH, MEPS and pain were the only explanatory variables that showed a significant correlation with QF improvement.

**Discussion** Our study pointed out that open surgery to treat elbow stiffness leads to significant improvement in patients’ clinical results and functioning and in their QF. Selecting the correct surgical procedure, one which is able to achieve a significant reduction in pain, appears to be the more relevant variable responsible for the improvement in QF. Finally, surgery shows a satisfactory CER, and this can justify an increase in health spending in this area, aimed at a reduction in the social costs resulting from lingering elbow stiffness.

**Conclusions** This study showed that the choice of adequate surgical technique is fundamental to obtain a good result. The first aim of surgery should be the reduction of pain, and then the recovery of elbow motion.

### Open surgical treatment of elbow stiffness: outcome in 41 patients

G. Giannicola, G. Bullitta*, F. Sacchetti, M. Scacchi, S. Carbone, F. Postacchini

“Sapienza” Università di Roma (Rome, IT)

**Introduction** The treatment of elbow stiffness is challenging for the surgeon. The purposes of this study were to evaluate the clinical outcome of open surgical treatment and the factors influencing outcome.

**Materials and methods** 41 patients (28 males, 13 females; mean age 48 years), treated by a single surgeon, were assessed. The elbow stiffness was caused by early degenerative arthritis in 6 patients, distal humeral non-union in 2, trauma in 31, and rheumatoid arthritis in 2. 17 Humeral-Ulnar Arthroplasty (HUA), 7 HUA associated to radiocapitellar replacement, 7 HUA associated to radial head prosthesis, 9 total elbow arthroplasty, and one HUA with anconeus interposition were performed. All patients had indometacin prophilaxis and started rehabilitation beginning from the second post-operative day. MEPS, m-ASES and DASH scores were used for outcome evaluation. The patient satisfaction was evaluated with a visual analogue scale (0–10).

**Results** The mean follow-up was 22 months (range 6–54). The average increase in MEPS and m-ASES was 44 (49–93) and 40 (45–86), respectively. The average decrease in Q-DASH and m-ASES pain was 42 (57–14) and 21 (22–43), respectively. The mean increase of flexion, extension, pronation and supination was 28° (105°–134°), 25° (40°–14°), 17° (63°–80°) and 15° (67°–82°), respectively. The differences observed were statistically significant. Four mild persistent ulnar paresthesia (one of which with a lack of strength of IV/V of the interosseous muscles), 1 deep infection, and 3 recurrent stiffness were observed. All but two patients were satisfied of surgical treatment. The average degree of satisfaction was 8.4 (range 4–10).

**Discussion** This study showed that open treatment of elbow stiffness allows obtaining a significant recovery of range of motion and a significant reduction of pain. A careful patient selection, a correct diagnosis, the choice of correct surgical technique and a proper rehabilitation protocol are four essential steps in the therapeutic algorithm that may affect the clinical outcome. Discussing and sharing with patients about possible risks and goals (frequently limited) of surgery is essential to obtain their satisfaction.
**Conclusions** This study confirmed the effectiveness of open surgical treatment of elbow stiffness.

### The safe zone for avoiding suprascapular nerve injury: an anatomical study on 500 dry scapulae

P. Albino*, M. Giaracuni, S. Carbone, F. Postacchini, S. Gumina

Dipartimento di Scienze dell'Apparato Locomotore,
Università di Roma "Sapienza" (Rome, IT)

**Introduction** Suprascapular nerve injury may be a complication during shoulder arthroscopy. Our aim was to verify the reliability of the existing data, assess the differences between scapulae in the two genders and in the same subject, obtain a safe zone useful to avoid iatrogenic nerve lesions, and analyze the existing correlations between the scapular dimensions and the safe zone.
**Materials and methods** We examined 500 dried scapulae, measuring six distances for each one, referring to the scapular body, glenoid and the course of the suprascapular nerve, also catalogued according to gender and side. Differences due to gender were assessed comparing mean ± SD of each distance in males and females; paired *t* test was used to compare distances deriving from each couple. Successively we calculated our safe zone and Pearson's correlation.
**Results** We found non-significant differences between the right and left distances deriving from each couple; differences due to gender were stated. We defined three kinds of safe zones referring to: 500 scapulae; males (139 scapulae) and females (147 scapulae). The correlation indexes calculated between the axis of the scapular body and glenoid and the posterosuperior distance (referring to the suprascapular nerve) were 0.624, 0.694, 0.675, 0.638; while those with the posterior distance were 0.230, 0.294, 0.232, 0.284.
**Discussion** We determined the posteosuperior limit (2.1 cm) and the posterior limit (1.1 cm) of the safe zone referring to the whole population. Gender influences the dimensions of the safe zone; the posterosuperior limit proved to be wider in males. The major axes of the scapular body and of the glenoid fossa are directly correlated to both the limits of the safe zone. The linear predictors, elaborated in this study, should be used to obtain specific values of the posterosuperior limit in each patient.
**Conclusions** The morphometric characteristics of the patient influence the dimensions of the safe zone. Gender and specific scapular dimensions should be evaluated since they influence the dimensions of the safe zone.

### Proximal ulnar and radial fracture-dislocation comprehensive classification system (PURCCS): diagnostic and therapeutic value in 25 cases

G. Giannicola, A. Greco*, D. Polimanti, F. Sacchetti, E. Manauzzi, M. Scacchi

"Sapienza" Università di Roma (Rome, IT)

**Introduction** Complex fracture-dislocations of the proximal ulna and radius include multiple anatomic lesions, difficult to manage and with often unpredictable result. Several classifications have been proposed; however, none of these appears to be exhaustive, and most of them have neither therapeutic nor prognostic value. Recently, we proposed a new comprehensive classification of these fractures based on specific pathoanatomic lesions, namely "cardinal lesions"; each of these is able to affect the prognosis and requires specific treatment. An alphanumeric system easy to remember and to use has been developed. Objective of this study was to evaluate diagnostic and therapeutic value of PURCCS in a series of patients.
**Materials and methods** We studied 24 patients (25 elbows) mean aged 57 years. All patients were classified using two standard radiographs, 2D and 3D CT scan and intra-operative fluoroscopy. According to "PURCCS", we observed: six type 5BIIICI, four type 2BIIICI, two type 3BIIICI, two type 1AI, one type 5BIII, one type 2BIIIE, one type 1BI, one type 5BIIICIII, one type 4AII, one type 1BIIICI, one type 1BIIICIII, one type 2BIIICID, one type 4AI, one type 5BIIICIIIE and one type 5BIIICID. Surgical treatment was performed according to the therapeutic algorithm of "PURCCS", briefly consisting on: anatomic reduction and stable internal fixation of all fractures, radial head replacement for irreparable fractures, repair of capsulo-ligaments lesions and hinged external fixator in presence of persistent instability. Indometacin prophylaxis and early rehabilitation were used in all patients. Clinical evaluation was performed according to MEPS.
**Results** The mean follow-up was 25 months. At last follow-up, the mean MEPS was 94. The functional ROM was obtained in 22 of 24 patients. Observed complications were: 1 ulnar nerve transitory palsy, 2 delayed cutaneous wound healings, 1 hardware failure and 2 stiffness. Each single pattern of fracture-dislocation observed in this series finds its own position within the PURCCS classification.
**Conclusions** All patterns of fracture-dislocation observed in our series were properly classified using PURCCS. The treatment algorithm suggested by this classification, allowed us to achieve excellent clinical results in 95 % of cases.

### Mid-term results of reverse shoulder arthroplasty

R. Castricini[1], G. Gasparini[2], M. De Benedetto[1], M. De Gori[2], O. Galasso*[2]

[1]Casa di Cura "Villa Verde" (Fermo, IT);
[2]Cattedra di Ortopedia e Traumatologia, Università degli Studi "Magna Græcia" di Catanzaro (Catanzaro, IT)

**Introduction** Reverse shoulder arthroplasty (RSA) is a successful procedure for the treatment of symptomatic glenohumeral osteoarthritis (OA) and cuff tear arthropathy (CTA). Short-term benefits of RSA have been previously reported, but few data are available with a longer follow-up. The purpose of this study is to assess the mid-term clinical and radiological results of RSA for CTA and primary OA, and to identify possible predictors of clinical outcome.
**Materials and methods** We prospectively evaluated 80 patients (59 women and 21 men, averaging 72.54 ± 5.03 years) that underwent RSA between 2003 and 2008 for CTA (58.75 %) or primary OA (41.25 %). Patients were pre- and postoperatively evaluated with Constant-Murley Score (CMS), range of motion, SF-36 and radiographic assessments.
**Results** After a mean follow-up of 60.09 ± 18.08 months, all clinical parameters improved from pre- to postoperative value (Wilcoxon $p < 0.001$ for all). In details, total CMS increased from 23.13 ± 7.29–66.36 ± 11.14, flexion increased from 100.13° ± 20.22°–150.25° ± 29.39°, abduction from 81.06° ± 18.55°–133.38° ± 34.92°, external rotation from 17° ± 10.51°–37.38° ± 16.76°, internal rotation from 47.06° ± 14.31°–63.63° ± 14.43°. Radiograms showed evidence of scapular notching and periprosthetic calcifications in 71 and 47 % of patients, respectively. Four patients (5 %)

developed complications; prosthesis dislocation occurred in 2 of them, and revision surgery was necessary.
**Discussion** Our results are consistent with those of previous reports on RSA after a shorter follow-up period, thus suggesting that clinical benefits after this procedure are maintained over time. We showed a complication rate of 5 % that is noticeably lower than the overall rate reported in previous studies.
**Conclusions** RSA achieves good results, with a low complication rate, in the treatment of primary OA and CTA, at least in the middle term. Future studies should confirm our findings in a longer observation time.

### *C04—KNEE 1*

### Tunnel enlargement CT evaluation: BPTB versus DSTG

R. Iorio, A. Redler*, G. Bolle, D. Mazza, P. Di Sette[1], F. Conteduca, A. Ponzo, A. Ferretti

Ospedale Sant'Andrea (Rome, IT)

**Introduction** Tunnel enlargement is a frequent issue after anterior cruciate ligament (ACL) reconstruction despite the type of graft used. The mechanism of bone tunnel enlargement following ACL reconstruction is not yet clearly understood. Previous studies showed how this phenomenon is more important after reconstruction with hamstrings (DSTG) than bone patellar tendon bone (BPTB). The purpose of our study was to prospectively evaluate the increase in size of the tibial tunnels following arthroscopic ACL reconstruction with either BPTB or DSTG autograft, using a CT scan.
**Materials and methods** Thirty-two patients surgically treated for ACL reconstruction were equally assigned to two different groups: group A (BPTB) or group B (DGST). Inclusion criteria were: male sex, age lower than 40 years, and chronic lesion of the ACL. The two groups were homogeneous for sex, age and activity level. Fixation devices used for patients of group A were nonabsorbable interference screws for tibial side; in group B we used a nonabsorbable interference screw with a metallic coil for the tibial side. Post-operatively, the rehabilitation protocols were different in the first month, with patients of group A starting the recovery of the range of motion the day after the surgery, and patients of group B 2 weeks later. All the patients started full weight-bearing at the same time, the day after the surgery. All the patients performed a CT scan the day after the surgery and at the follow-up (13 months: range 11–15 months). The measurements were performed following a radiological protocol previously published. Statistically analysis was performed using the *t*-Student test.
**Results** In group A (BPTB group) CT images showed an average increase in diameter of the tibial tunnel from 9 mm to 12.2 ± 1.85 mm (range 10.0–14.3 mm) ($p < 0.01$). In group B (DSTG group) the mean diameter of the tibial tunnel increased from 9 to 10 mm ± 0.8 mm (range 9.2–11.6 mm), ($p < 0.01$). The difference between the two groups was statistically significant ($p < 0.01$).
**Discussion** Differently from other similar studies, this paper is the first to describe a higher enlargement of tibial tunnels in patients operated on for ACL reconstruction with BPTB. We could speculate that this result might be due to a stronger stiffness of the fixation devices used in DSTG group and to a different rehabilitation protocol used.
**Conclusions** ACL reconstruction with DSTG with stronger and stiffer biomechanical properties of the fixation devices along to a slower rehabilitation protocol seems to be effective in reducing tibial tunnel enlargement.

### Partial versus complete anterior cruciate ligament reconstruction

L. Gala*[1], F. Uboldi[1], G. Vergottini[1], E. Usellini[2], B.M. Marelli[2], W. Albisetti[3], C. Mazzola[4], M. Berruto[2]

[1]Scuola di Specialità in Ortopedia e Traumatologia, Università degli Studi di Milano (Milan, IT);
[2]Istituto Ortopedico G. Pini (Milan, IT);
[3]Università degli Studi di Milano (Milan, IT);
[4]E.O. Ospedali Galliera (Genoa, IT)

**Introduction** The anterior cruciate ligament (ACL) has two functionally separate and distinct bundles: one is antero-medial (AM), and the other is postero-lateral (PL). This finding allowed us to improve the ACL reconstruction surgeries from a single bundle technique to a double bundle one. Therefore this evolution opened the way to a partial reconstruction technique, widely described in literature in recent times.
**Materials and methods** Between January 2009 and February 2011 we performed a partial ACL reconstruction in 55 patients (37 males and 18 female) with a mean age of 32 years (range 17–55), which suffered from a partial ACL tear. In 49 cases we reconstructed the AM bundle and in 6 cases the PL. In one patient we performed a ACL revision surgery reconstruction only the AM bundle because the PL was still intact. For the AM bundle reconstruction we used: 34 doubled semitendinous (ST) ligaments, 2 "mini" patellar tendons (BPTB), 4 doubled gracilis (GR) tendons and 1 doubled allograft ST. For the PL bundle we used 2 doubled ST, 1 doubled GR and 1 Achilles ligament allograft. For the femoral fixation we used 46 times a suspension technique, 7 times a cross pins fixation and in 2 cases was fixed with reabsorbable screws. The fixation on the tibial side was performed in 8 cases with reabsorbable tapers and screws and in 35 cases with reabsorbable screws. Within this study we evaluated 49 patients with a mean follow-up of 20 months (range 12–38). All the patients were evaluated objectively, functionally and subjectively with: IKDC subjective and functional, Tegner, Lysholm knee scale, KT1000 and KiRA.
**Results** In more than 90 % of the cases the results were between excellent and good functionally, objectively and subjectively.
**Discussion** The results obtained were then compared with 2 homogeneous groups of 49 patients each (Group I and II) that underwent in the same span of time a complete ACL reconstruction with ST-GR or BPTB.
**Conclusions** The Group I compared to Group II and III showed better functional results and faster return to sport activity.

### *C05—SPORTS TRAUMATOLOGY*

### The FIFA 11+ program for the prevention of injuries in basketball: a cluster randomized controlled trial

U.G. Longo[1], M. Loppini*[1], A. Berton[1], A. Marinozzi[1], N. Maffulli[2], V. Denaro[1]

[1]Università Campus Bio-Medico di Roma (Rome, IT);
[2]Queen Mary University of London (London, UK)

**Introduction** In the last years, structured training programs for sports injury prevention (''The 11'' and ''The 11+'') have been validated in soccer. However, the FIFA 11+ program has not been evaluated in basketball. The aim of the study was to assess the effectiveness of the FIFA 11+ program in preventing injuries in male basketball players.
**Materials and methods** We randomized 11 teams of the same club, allocating seven teams to the intervention group (80 players; 13.5 ± 2.3 years), and 4 teams to the control group (41 players;

15.2 ± 4.6 years). During a 9-months season (August 2009 and April 2010), we conducted an injury surveillance program. The primary and secondary outcomes were any injury to the athletes and any injury to the lower extremity respectively. We also performed an analysis of the type of exposure (match or training), injury location in the body, and type of injury (acute or overuse).

**Results** 23 (19 %) of the 121 players included in the study sustained a total of 31 injuries (14 in the intervention group and 17 in the control group). In the intervention group, injury rates per 1,000 athlete-exposures were lower than those in the control group, with statistical significance, for overall injuries (0.95 vs. 2.16; $p = 0.0004$), training injuries (0.14 vs. 0.76; $p = 0.007$), lower extremity injuries (0.68 vs. 1.4; $p = 0.022$), acute injuries (0.61 vs. 1.91; $p < 0.0001$), and severe injuries (0 vs. 0.51; $p = 0.004$). The intervention group also had statistically significant lower injury rates for trunk (0.07 vs. 0.51; $p = 0.013$), leg (0 vs. 0.38; p = 0.007), and hip and groin (0 vs. 0.25; $p = 0.023$) compared with the control group. There was no statistically significant difference in match injuries, knee injuries, ankle injuries, and overuse injuries between 2 groups. The most frequent acute injury diagnoses were ligament sprains (0.41 and 0.38 in the intervention and control groups, respectively; $p < 0.006$) and contractures (0.76 and 0.07 in the control and intervention groups, respectively; $p < 0.003$).

**Discussion** In soccer, the FIFA 11+ was able to prevent overall injuries, knee injuries, lower extremity injuries, severe injuries, and overuse injuries. In our population, it reduced the risk of trunk, leg, and hip/groin injuries, lower extremity injuries, overall injuries, and severe injuries. Although, the rate of knee and ankle injuries was not reduced, the FIFA 11+ was able to reduce the severity of such injuries.

**Conclusions** The FIFA 11+ warm-up program is effective in preventing injuries in elite male basketball players.

### Refractory tendinopathies treatment with echo-guided infiltration of PRP

F. Rosso[1], E.C. Cenna*[2], A. De Marchi[3], S. Pozza[3], G. Gays[1]

[1]II Clinica Ortopedica, CTO-M. Adelaide, Università degli Studi di Torino (Turin, IT);
[2]II Clinica Ortopedica, CTO-M. Adelaide (Turin, IT);
[3]Dipartimento di Radiodiagnostica, CTO-M. Adelaide (Turin, IT)

**Introduction** Chronic tendinopathies are difficult to treat an they often do not respond to treatment. Some Authors suggested that Platelet Rich Plasma (PRP) was able to stimulate reparative process. Aim of this study is to correlate clinical and echographic effectiveness of echo-guided intra-tendinous infiltration of 3 doses of PRP at a distance of 2 weeks at a concentration of 6 ± 2 millions of platelets at each inoculum.

**Materials and methods** Inclusion criteria were chronic tendinopathies (Jumper's knee, epicondilitys, Achille's tendinopathy) refractory to conservative therapies in patients engaged in amatorial sports. Patients affected by diabetes, cardiovascular disease, sepsis and immunodepression were excluded. 3 PRP intra-tendinous echo-guided injections were performed at a 15 days' distance. All patients were radiologically studied pre-treatment and at 6 months with US, Doppler US with contrast, and MRI, and clinically evaluated using international scores (Tegner, Womac, VAS and EQ). 11 patients reentry our study with an average age of 43.2 years (SD ± 14.3). For statistical analysis, *t* Test was performed, and it was correlated with average and standard deviation.

**Results** No complications correlated to the procedure were found. All the scores increase from pre-treatment, but not all had a statistically significant improvement. In particular, Tegner and EQ did not have a statistically significant increase (respectively $p = 0.675$ and $p = 0.502$); on the contrary VAS and Womac had a statistically significant improvement (respectively $p = 0.0344$ and $p = 0.0009$), 3 patients required surgical intervention for the persistence of symptoms and therefore were considered as failures. At 6 months post-treatment radiographic analysis there was a reduction of 85 % in microcalcifications.

**Discussion** Using this technique we had good clinical and radiographic results. In some cases at post-treatment MRI the almost complete remission of oedema and intratendinous calcifications was found. Actually we proceed with this study with the aim to increase the casuistry.

**Conclusions** We believe that improvement in VAS and, overall, in WOMAC are encouraging. Besides also echographic results are encouraging for what it concern pathologic morphology, oedema, intratendinous microcalcification and pathologic vascularization reduction.

### Anterior cruciate ligament reconstruction with LARS® artificial ligament: review and results at medium-term follow-up

P. Parchi*, A. Baluganti, L. Dolfi, G. Ciapini, F. Chiellini, M. Lisanti

I Clinica Ortopedica Universitaria (Pisa, IT)

**Introduction** During the last years, as result of the development of new biomaterials and more accurate surgical techniques, the interest about the possibility to use synthetic grafts for the reconstruction of the ACL is newly grown. The LARS® artificial ligament has recently been reported to be a suitable material for ACL reconstruction.

**Materials and methods** Aim of this study is to review the patients that underwent ACL reconstruction with LARS® ligament in the 1st Orthopaedic Department of Pisa University during the period between January 2003 and December 2006 to evaluate the safety and efficacy of the treatment and its actual indications. These surgical operations were carried out in patients older than 30 years with symptomatic ACL lesions. All patients were strongly motivated by work or sport requirements and needed a fast functional recovery. Fifty patients were reviewed with a mean follow-up of 77.6 months. The review protocol was articulated in 3 phases: (1) a subjective evaluation using 3 grading scales VAS, KOOS and Cincinnati Knee rating scale; (2) a clinical and radiographic evaluation; (3) a biomechanical evaluation of the knee stability and proprioception.

**Results** From the subjective evaluation, we obtained a 90 % of positive results (average Cincinnati score 85.8—average KOOS 90.1) with a return to the previous activity level after an average of 3.1 months. The average VAS value was 2.1. There were no cases of reactive synovitis or knee infection. We recorded only a case of mechanical graft failure that occurred during a high level sport activity. The results obtained from the knee stability evaluation were positive in 93 % of the patients. The stabilometric analysis showed that there was not significant statistical difference in knee proprioception between the operated knee and the contralateral one.

**Discussion** The main advantages of using LARS® ligament are the absence of the donor site morbidity, the patient's fast functional recovery and the absence of major complications (no cases of synovitis was observed in our series). Our data are in agreement with the literature data where only very rare cases of knee synovitis are reported.

**Conclusions** We conclude that the LARS® ligament can be considered, in selected cases, a suitable option for ACL reconstruction especially for patients that need a fast functional recovery. The results obtained from the patients review were very encouraging and similar to the literature data; however to completely asses the long term safety and efficacy of the LARS® further long term studies are needed.

### Treatment of tendon lesions with or without platelet rich fibrin

M. Valeo, M. Giaracuni, P. Persiani, R. Lanzone, G. Mazzotta*, L. Sodano, L. Di Martino, M. Gurzì, L. Di Sante, C. Villani

Dipartimento di Scienze dell'Apparato Locomotore, Policlinico Umberto I, Università di Roma La Sapienza (Rome, IT)

**Introduction** To date the role of PRP in tissue repair is still debated by many authors. Its importance, therefore, is not clarified and its applications are limited. Different authors use the PRP within the joints in order to soothe the pain and as an analgesic, other authors suggest (although in literature there is a lack of comprehensive and significant studies) that the PRP has a role in chondroprotection or chondro-repair. The largest number of studies instead have demonstrated the effectiveness of PRF to accelerate the repair of myotendinous injury and at the same time ensure an early recovery of joint function thanks to the decrease or even the absence of pain.
**Materials and methods** We compared the long-term outcomes of 4 types of patients in which the PRF is also used: a quadriceps tendon and Achilles tendon treated open with traditional technique; a patellar tendon with chronic rupture and proximally dislocation of patella treated with traditional repair and semitendinosus augmentation; a plantar fascia detached from calcaneal joint not treated surgically but with ultrasound-guided infiltration of PRP. We have also evaluated the same lesions in as many patients treated in the same way, but without the use of PRF/PRP. The evaluation was performed taking into account the medical record and the mobilization by the physiotherapist, a VAS scale proposed for patients in the seventh postoperative day and the healing of the wound in the fifteenth day. Were also performed instrumental examinations at a distance of time to evaluate the post-surgery course.
**Results** Our patients treated with PRP had a VAS score with an average of 2–3 in spite of those who have not used the PRP which instead had a VAS scale of 6–7, this has enabled patients with minor painful symptoms to address early and better post-operative rehabilitation and to obtain a quicker recovery. However, with respect to the healing of the wound there were not significant evidences in the orthopaedic field unlike the plastic and maxillo-facial surgery, instead widely discussed in the literature.
**Conclusions** In accordance with the worldwide scientific literature, to date we are not yet able to say that the PRF/PRP might have an essential role in tissue repair. But we can say that patients, treated with traditional surgery and in addition the application of PRF or PRP, have less debilitating pain that also allows approaching to an earlier rehabilitation.

### *C06—HIP 2*

### Treatment of hip osteonecrosis with regenerative medicine and minimally invasive surgical technique

T. Frisoni*, L. Cevolani, B. Spazzoli, B. Dozza, E. Lucarelli, D.M. Donati, S. Giannini

Istituto Ortopedico Rizzoli (Bologna, IT)

**Introduction** Avascular osteonecrosis of the femoral head is a disease that predominantly affects subjects between 20 and 40 years. When it is not a consequence of trauma, is associated with steroid use, alcoholism, storage diseases, coagulopathies, hematologic and autoimmune diseases, but a considerable fraction of osteonecrosis was idiopathic. If left untreated, it leads to the collapse of the femoral head with severe functional limitation and early progression to osteoarthritis. There are several possible approaches with mixed results.
**Materials and methods** Since September 2008 we treated 38 osteonecrosis in 36 patients (28 males, 8 females) mean age 36 years (min 17-max 64) with core decompression of the necrotic area by transtrochanteric approach in minimally invasive surgery and application of concentrated autologous bone marrow, autologous platelet gel and demineralized bone matrix. In 13 patients the necrosis was idiopathic, in 19 high-dose steroid use related, post traumatic in 5 patients, alcohol abuse in one patient. We used the Ficat classification to stage the degree of necrosis: in 24 cases was found to be stage II (15 cases IIa, IIb 9 cases), while in the remaining 14 cases necrosis was stage III-IV (11 cases III, 3 cases IV). The outcome was assessed using Harris Hip Score (HHS), x-rays and MRI in 45 days, 3, 6, 12 and 24 months and the treatment was considered failed if a prosthetic replacement was necessary.
**Results** The average follow-up was 11 months (min 45 days-max 33 months).The HHS has shown an increase (from 59 to 84); the patients with Ficat stage II of the necrosis have shown a better clinical response (from 60.4 to 84.5) than patients with stage III-IV (56.7–83.5). In two cases the treatment failed.
**Discussion** Local conditions that lead to osteonecrosis require a treatment that stimulates tissue regeneration while preserving the integrity of anatomical structures. The rationale of our method is to improve the local regenerative microenvironment, providing the stimulus (decompression and growth factors) and osteoblastic precursors, with a minimally invasive technique which does not affect the joint vascularization, leading to clinical and radiographic good results.
**Conclusions** The core decompression of the necrotic area associated with application of concentrated autologous bone marrow, autologous platelet gel and demineralized bone matrix is a good alternative to other salvage therapies to prevent femoral head prosthetic replacement. The clinical and radiographic results are satisfactory and promising, although they must be considered preliminary.

### Three-dimensional MRI study of the hip prosthesis

E. Bonicoli*[1], V. Zampa[2], L. Andreani[1], N. Piolanti[1], M. Lisanti[1]

[1]Ortopedia e Traumatologia I (Pisa, IT);
[2]Radiodaiagnostica I Universitaria (Pisa, IT)

**Introduction** The main indication for MRI is the painful prosthesis that does not show abnormalities on X-ray images. MRI may also be useful in the diagnosis of infection, periprosthetic tendonitis, bursitis, periprosthetic osteolysis evaluation and early bone marrow changes. The purpose of this study is to evaluate muscle–tendon "alterations" occurring after hip replacement using a particular three-dimensional MRI sequence.
**Materials and methods** At the Orthopaedic I Department and Radiodiagnostic I Department of the University of Pisa, from June 2010 to December 2011, 14 patients were submitted to total hip prosthesis and monitored with MRI. All patients underwent clinical and X-ray evaluation, Harris Hip and WOMAC scores; preoperative, 1 week, 3 and 12 months post-operatively MRI was performed. Surgery was performed by the same surgeon with standard postero-lateral approach and a posterior soft tissue repair was done. All MRI studies were evaluated by one experienced radiologist. MRI was performed with a 1.5 T system using a phased-array coil (8 channels). Multivolumetric reformatting was obtained in order to better depict the posterior pseudo-capsule gap, fluid collection, and any fat atrophy of the muscles.
**Results** The pre-surgical diagnosis was dysplasia (n = 3), aseptic necrosis (n = 2) and osteoarthritis (n = 9); 6 m, 8f, mean age

59 years. In particular, a posterior pseudo-gap greater than 5 mm and a mild fat atrophy of the obturator internus muscle was found in 2 patients and it persisted in the 3 months follow-up MRI. The quadratus femoris remained intact in 13 patients (97 %) and in 6 patients there was no gap between the great trochanter and piriformis tendon signal (43 %).

**Discussion** Thanks to the high spatial resolution and the possibility to follow the anatomy of the musculo-skeletal structures of the 3D sequences, the gap as well as the attachment of the tendons resulted to be more easily detected than on conventional imaging. In our series we did not find any significant correlation between clinical out-come and radiological findings, just for 1 patient. In this case the MRI images were useful to correlate groin pain with adductor attachment oedema.

**Conclusions** Based on our experience, the possibility to visualize in a three dimensional way enables a more accurate assessment of the post-surgical modified anatomy of the periarticular structures. Further studies are needed to correlate the modified anatomy with post surgical symptoms. Despite the long scan time, the 3D sequence offers an optimal anatomical depiction and can replace the standard MRI sequences for this exam.

### *C07—TRAUMATOLOGY 2*

### The management of surgical acetabular fractures: considerations on a personal series

G. Topa*[1], P. Ditto[2], S. Barreca[2], A. Fazzalari[2], M.A. Rosa[2]

[1]UO di Ortopedia e Traumatologia, Azienda Ospedaliera Bianchi-Melacrino-Morelli (Reggio Calabria, IT);
[2]Scuola di Specializzazione in Ortopedia e Traumatologia, Sezione di Ortopedia e Traumatologia, Dipartimento Specialità Chirurgiche, Università degli Studi di Messina (Messina, IT)

**Introduction** The acetabular fractures are a problem of major importance in traumatology, either for the difficult classification, as for complex surgical managing which requires qualified and equipped centers. There are two basic principles for treatment of these types of fracture: operation must be performed within 3 weeks and the chosen surgical technique must provide an anatomic and stable reduction. The most involved patients are young adults, with a higher incidence in males than females. The main causes are road accidents (dashboard injuries) and falls from above. These fractures often are associated with fractures of pelvis and femur. Mortality is especially high in polytrauma (10 %).

**Materials and methods** Between January 2007 and December 2011 at the Ospedali Riuniti of Reggio Calabria 28 patients with fractures of the acetabulum were treated; all patients were preoperatively studied by X-rays (AP, and span obturator) and CT with 3D reconstruction; the trans-skeletal traction has been applied to all patients, for the period between the trauma and day of surgery (on average 3 days). Classifications used are the Judet-Letournel simplified one, the Tile's one and the AO's one.

**Results** The 28 cases are classified: 7 transverse fractures, 8 fractures of the back wall, 5 bi-columnar fractures, 3 fractures of the posterior column, 3 fractures of the anterior column and 2 T—fractures. In 40 % of cases (11) the dislocation of the hip was also associated; all patients were treated with ORIF synthesis. The results were evaluated by testing post-operative X-rays at 1-3-6 and 12 months.

**Discussion** The used surgical approaches were the Kocher-Langenbech for fractures of the posterior wall, fractures of the posterior column and associated fractures of the posterior column and posterior wall; the ileo-inguinal approach was used in fractures of the anterior wall or fractures of the front column and associated fractures of column and front wall. In bi-columnar and transverse fractures a dual approach was performed. The most feared complications, in addition to infection and thromboembolism, are: immediately hip dislocation/subluxation; aseptic necrosis of the femoral head at long-term and the inevitable post-traumatic arthrosis.

**Conclusions** Cases where an anatomical reduction is obtained, with less than one mm diastasis, were considered excellent; cases where the diastasis was within 3 mm were considered sufficient. Bad results have occurred in 10 % of cases. Conservative treatment can give good results only in very selected cases.

### Diagnosis and management of unstable pelvic fractures

N. Torelli*[1], R. Politano[2], A. Longhi[2], L. De Palma[1]

[1]Clinica Ortopedica (Ancona, IT);
[2]U.O. Ortopedia, Ospedali Riuniti (Ancona, IT)

**Introduction** The pelvis fractures which are caused by high-energy trauma, have serious and complex prognosis, especially in relation to the bleeding risk. New treatment methods for the damage control of polytrauma patients require different skills. A decision-team for the patient management is necessary. In literature there is not uniform indication for the management, the diagnostic and the treatment of this kind of patient. We aren't able to apply a single algorithm at the treatment of this complex trauma because a high frequency of different lesions is associated with this kind of fractures.

**Materials and methods** This study is a retrospectively review. We admitted 71 polytrauma patients. They arrived to DEA of II level at the Ancona Hospital from 2006 to 2010. Inclusion criteria were: major trauma and simultaneous unstable pelvic fracture. Excluded criteria were: girdle pelvic fractures composed or stable; hip fractures without acetabulum fracture or iliac wing fracture. We evaluated the diagnostic and therapeutic pathways and defined the role of external fixation and angiography with possible embolization. Finally, we described the surgical stabilization definitive mode, in relation to the different kinds of fractures.

**Results** The most common algorithm is based on the diagnostics in the emergency room, followed by angiography and embolization when possible and anterior external fixation in patients with unstable pelvic fractures. Exploratory laparotomy was performed when the hemoperitoneum was present. The definitive treatment takes advantage of the use of plates, screws and external fixators in according to the kind of fracture.

**Discussion** The retrospective review allowed to asses our algorithm. The statistical analysis showed that the immediate damage control and the multidisciplinary management reduces mortality and optimizes the polytrauma patient management (early treatment in emergency, reduces transfusions and accelerates the discharge).

**Conclusions** The association between external fixation and angiography is the most common solution in controlling the arterial and venous bleeding, and fracture bleeding in the damage control of polytrauma patients with unstable pelvis fractures.

### Our experience in the treatment of humeral fractures with polarus nail

R. Ciarla*, G. Bertoni, D. D'antona

Struttura Complessa di Ortopedia Traumatologia, Ospedale di Treviso "Cà Foncello", Regione Veneto, ULSS 9 (Treviso, IT)

**Introduction** Polarus nail (Standard and Plus) is employed in the treatment of the shaft humeral fractures and third proximal, often associated to the proximal humeral epiphyseal fractures to 2 and 3 fragments. The purpose is to supply to the surgeon an excellent stability of the fracture through a mini-invasive procedure, an adapted fixation in osteoporotic bone, to avoid the axillary nerve, to reduce the fracture risk around the device, to supply an efficient and reliable guidance system, to avoid excessive distal dissections through the guide, to supply different options for various types of the fracture.
**Materials and methods** It is brought back a monocentric survey that includes 280 cases operated in between 2001–2011; in 162 cases the nail in the Plus version was employed, in 112 cases the nail in Standard version and in 6 cases a retrograde assembly was executed; in 3 cases a bilateral assembly was carried out.
**Results** 50 patients were revaluated at distance. The recovery of the fracture was observed in 49 cases, while we observed 1 case of pseudoarthrosis. Neither sepsis nor neurological complications were detected.
**Discussion** We show the more meaningful and discussed cases and the adopted therapeutic indications.
**Conclusions** After all, the device in use resulted manageable and reliable with "a learning curve" relatively short.

### *C08—SHOULDER AND ELBOW 2*

### The bCAT technique: a new solution to restore tuberosity position and cuff tension in reverse shoulder prosthesis for complex proximal humeral fractures

F. Cautiero*, R. Russo, M. Ciccarelli, G. Giudice

U.O.C. di Ortopedia e Traumatologia, Ospedale dei Pellegrini (Naples, IT)

**Introduction** The reverse shoulder prosthesis is an alternative treatment proposed in the elderly patients with a complex proximal fracture but the results are controversial with poor functional outcomes especially for restoring the extrarotation and abduction of the shoulder. We propose a new technique, "bCAT" (bone Collar And Tie) to apply in reverse prosthesis improving tuberosities healing and shoulder function.
**Materials and methods** Thirty-four patients with a mean age of 73.8Y (range: 68–95) were treated with a reverse prosthesis between 2005 and 2010 for a complex proximal humeral fracture. In 30 shoulders we used a cementless reverse prosthesis SMR (Lima lto). In four patients we implanted a cemented reverse prosthesis: two SMR and two Delta-DePuy (Johnson&Johnson). In 18 cases we carried out the new surgical technique called "bCAT" that realize using the fractured humeral head, adequately modelled, a collar neck-tie for increase the mechanical and biological support to improve the healing of tuberosities and functional results.
**Results** The results are evaluated in twenty patients divided in two groups: 10 patients treated with standard surgical procedure (group A) and 10 patients in which we used the bCAT technique (group B). In the group A the average range of motion was 109.4° for abduction, 125° of anterior elevation, 15° of the extrarotation in RE1 and in intrarotation the hand reached the Lumbar-Sacrum area. The mean pondered Constant Score was 64.7. In the group B the mean abduction was 138°, mean elevation was 160°, mean extrarotation in RE1 was 35° and in intrarotation the hand reached second or third Lumbar spine. The mean pondered Constant score was 84 %.
**Discussion** Tuberosity position and healing is critical for good outcomes in the shoulder trauma arthroplasty. In particular the rate of resorption of the tuberosities in anatomical and reverse trauma prosthesis is still elevated and clinical results particularly concerned the rotation are in the most cases unsatisfactory.
**Conclusions** This new technique could be increasing the clinical and radiological results in the reverse prosthesis, improved the healing of tuberosities.

### The predictivity of AVN in proximal humerus fractures in accordance with the Hertel's criteria

G. Campochiaro*, M. Rebuzzi, C. Rovesta, F. Catani

Azienda Ospedaliera Universitaria Policlinico (Modena, IT)

**Introduction** The purpose of this study is to assess applicability and reproducibility of the Hertel's criteria about the predictivity of AVN in a cohort of proximal humerus fractures treated with plate at our Center and followed with seriated X-rays.
**Materials and methods** 267 intracapsular proximal humerus fractures treated with angular-stability plate between January 2004 and December 2010. We have managed a retrospective X-ray examination study, calculating for each patient on the fracture X-ray or CT-scan (if present) those parameters that were considered predictors of AVN by Hertel. Moreover we have assessed the presence or absence of head ischemia at the last X-ray performed. These criteria are subdivided into good (calcar <8 mm; disrupted medial hinge; fracture type 2-9-10-11-12 by LEGO classification—with 3 classifiers) and moderate/poor predictors (head angular displacement >45°; greater tuberosity displacement >10 mm; gleno-humeral dislocation; impression-fracture; head-split). The presence of ischemia was classified according to Cruess (CORR, 1978). The statistical analysis was conducted by SPSS 18.0®.
**Results** Evaluation included 267 patients: 73 % F, 27 % M; average age 65 years; 70 fractures CT-scan (26.2 %); mean follow-up 18.2 months (min 6.2-max 73.9); average fracture-surgical treatment time 5.7 days. AVN was 10 (3.7 %): 70 % F; 60 % > 65 years. In 7/10 fractures the anatomic reduction quality of the surgical treatment was good/poor, and only in 4/10 the reduction was maintained at follow-up. Good predictors of ischemia was present in 30 % (3/10), 1 of these 3 cases presents also angular head displacement >45° and greater tuberosity displacement >10 mm (10 %), no gleno-humeral dislocation and head-split contemporaneously. In 257 (96.3 %) fractures without AVN, 12 patients had 3 good predictors of ischemia (4.7 %), 3/12 showed also greater tuberosity and head angulation displacement (1.2 %), no association with gleno-humeral dislocation and head-split contemporaneously.
**Discussion** The application of the Hertel's criteria at the diagnosis step of these type of fractures, nowadays seems essential for a correct therapeutic iter. Anyway, the fracture evolution in AVN can follow biological criteria sometimes not clear.
**Conclusions** The importance of the predictivity of the Hertel's criteria was confirmed by our data. Nevertheless, some of these fractures evolve in AVN without an evident correspondence within these criteria. To conclude, we have to discover biological parameters not well-known. The data statistical analysis shows that this study will have more account with an average larger follow-up.

### Optimal positioning of the humeral component in the total inverse shoulder prothesis

L. Nalbone*[1], R. Adelfio[1], M. D'arienzo[1], F. Campi[3], P. Paladini[3], A. Pellegrini[3], G. Porcellini[3], V. Nigrelli[2], F. Zabbara[2], T. Ingrassia[2]

[1]Clinica Ortopedica e Traumatologica del Policlinico “Paolo Giaccone” dell’Università degli Studi di Palermo (Palermo, IT);
[2]Dipartimento di Ingegneria Chimica, Gestionale, Informatica, Meccanica, Università degli Studi di Palermo (Palermo, IT);
[3]Laboratorio di Biomeccanica, Ospedale Cervesi di Cattolica (Cattolica, IT)

**Introduction** Total inverse shoulder replacement is becoming more and more the standard therapeutic practice for gleno-humeral arthritis with massive lesions of the rotator cuff. The mobilization of the glenosphere represents one of the most frequent causes of clinical complications in this type of implant. The stability and the duration of the attachment of the new implant to the scapula depends on: bone quality, screw pressure, the positioning and the lateral movement in relation to the glenoid surface, the wear of the polyethylene and the load metaphisis on the stem. The present work aims at studying the effect of the positioning of the humeral component on the stability of the glenoid component. In particular, we want to establish the quantity of the loads transmitted to the glenoid component of the prosthesis and discharged on to the scapula according to the variation in the version angle of the humeral component.
**Materials and methods** The CAD model of the prothesis is created with the data obtained using the 3D scanner and the parts are assembled. In the second phase a numerical analysis is carried out using the finite element method (FEM). The attributed numerical code allows the breaking down of the continuous model into a high number of elements of finite dimensions and the discovery of the state of tension on the components when the geometric parameters and the load conditions vary.
**Results** Some static configurations are implemented in order to determine the extent of the loads transmitted to the glenoid component of the prosthesis and unloaded on to the scapula when the version angle of the humeral component varies. The best position of the humeral component is found together with the loads transmitted and the relative tensions corresponding to that condition.
**Discussion** The analyses of the finite elements, carried out using the model obtained with the 3D scanner, have allowed the calculation of the loads transmitted and the relative tensions corresponding to the position that minimizes the state of stress of the humeral component, so as to reach clinical considerations.
**Conclusions** The Authors have studied the influence of the positioning of the humeral component on the stability of the glenoid component of the inverse shoulder prosthesis. They determined the entity of the loads transmitted to the glenoid component of the prosthesis and unloaded on to the scapula when the version angle of the humeral component of prosthesis varies, using FEM numerical analysis.

### *C09—KNEE 2*

### Conservative treatment of early stages of the non-traumatic avascular necrosis of the knee with pulsed electromagnetic fields: a 2-year minimum follow-up study

G.M. Marcheggiani Muccioli*[1] A. Grassi[1], E. Rimondi[1], L. Zambelli[2], A. Scarale[1], A. Russo[1], G. Filardo[1], S. Zaffagnini[1], M. Marcacci[1]

[1]Istituto Ortopedico Rizzoli (Bologna, IT);
[2]Villa Igea Hospital (Forlì, IT)

**Introduction** The aim of the present study is to investigate the effect of Pulsed ElectroMagnetic Fields (PEMFs) treatment in patients suffering from early stages of spontaneous avascular necrosis (AVN) of the knee.
**Materials and methods** Twenty-eight pts (19 M, 9F, mean age 49.8 ± 16.4 years, BMI 26.0 ± 3.9) suffering from spontaneous AVN of the knee were prospectively enrolled in this study. Inclusion criteria: presence of a sympthomatic (acute and progressive pain) spontaneous AVN of the knee (a non-traumatic Bone Marrow Lesion (BML) of this joint [2]) in a middle-aged or elderly patient, without ligamentous knee laxity. Exclusion criteria: previous knee surgery or presence of total hip replacement on affected or contra-lateral limb, varus or valgus knee deformity exceeding 10°, BMI > 30 kg/m$^2$, infection, rheumatoid arthritis, autoimmune diseases, systemic diseases, tumours, use of steroids, alcohol or nicotine abuse. The patients were treated with PEMFs (I-ONE-therapy-IGEA: field 1.5 mT, frequency 75 Hz, duty-cycle 10 %) for at least 6 h daily for 1 month. Clinical evaluation: time zero, 6, 12 and 24 months after treatment by means of VAS for pain, Knee Society Score (KSS), Tegner and EQ-5D scales. MRI studies were also performed at time zero and 6 months, measuring BMLs areas (by Osirix-MD-Software) and grading these lesions by means of WORMS score [3]. Failures (patients undergoing unicompartmental or total knee arthroplasty) were recorded until 2-year follow-up.
**Results** Pain was significantly reduced after 6 months (mean VAS from 73.2 ± 20.7–29.6 ± 21.3; $p < 0.0001$). Clinical scores significantly improved at 6 months: mean KSS (from 34.0 ± 13.3 to 76.1 ± 15.9; $p < 0.0001$), median Tegner (time zero 1, range 1–1; follow-up 3, range 3–4; $p < 0.0001$) and mean EQ-5D (from 0.32 ± 0.33 to 0.74 ± 0.23; $p < 0.0001$). MRI evaluation showed a significant reduction of BMLs areas for femoral lesions, especially in the sagittal plane ($p < 0.005$). This area reduction is strongly correlated to WORMS grading ($p < 0.005$). Four patients required a knee arthroplasty (2 unicompartmental and 2 total). PEMFs therapy preserved 85.7 % of treated knees.
**Discussion** PEMFs stimulation was able to significantly reduce knee pain and to increase knee function. It was able to preserve 85.7 % of the knees from prosthetic surgery a 2-year minimum follow-up. These results are comparable to those obtained by Massari et al. [1] in the AVN of the femoral head.
**Conclusions** The results of this study confirmed that PEMFs can have a role in the treatment of early stages of spontaneous AVN of the knee. However randomized controlled studies are needed on this topic.

**References**

1. Massari (2006) JBJS Am 88:56–60
2. Roemer (2009) Osteoarthritis Cartilage 17:1115–1131
3. Peterfy (2004) Osteoarthritis Cartilage 12:177–190

### Rotating hinge prosthesis in first implants: a comparison of two models

F. Rosso*[1] A. Bistolfi[2], M. Paonessa[2], L. Drocco[2], A. Cimino[1], M. Crova[3], G. Massazza[3]

[1]Università degli Studi (Turin, IT);
[2]AO CTO M. Adelaide (Turin, IT);
[3]AO CTO M. Adelaide, Università degli Studi (Turin, IT)

**Introduction** Many authors suggest that hinged prosthesis are linked to an increased risk of aseptic loosening because of higher stresses transmitted to bone-prosthesis or bone-cement interface. Aim of this study was clinical and radiographic evaluation of two different rotating hinge prosthesis used in first implant.
**Materials and methods** From 1995 to 1995, 98 Endo-Model (Link®) were implanted in patients with an average age of 69.1 years

(SD ± 7.4) and in 68.4 % in cases of knee arthrosis due to axial defects (Group E). From 2002 to 2008 12 RHKnee (Zimmer®) were used in patients with an average age of 55.1 years (SD ± 12.7) and in 33.3 % in cases of knee arthrosis due to axial defects (Group R). Hospital-Special-Surgery questionnaire was used for clinical evaluation and "Knee Society Roentgenographic Evaluation System" for radiographic ones. Data were collected in a prospectively; *t*-Student test was used for statistical analysis and cumulative survivorship was calculated using Kaplan-Maier's method.

**Results** Group E follow-up was 174.1 months; Group R was 43.7 months. All HHS scores showed a statistically significant increase in both groups. In particular, range of motion increased from 88.2° (SD ± 7.6°) to 109.5° (SD ± 17.3°) in Group E, and from 97° (SD ± 23.9°) to 115° (SD ± 13.8°) in Group R. In Group E there was 18 failures (25 %), of which 11.1 % of aseptic loosening; 15 years cumulative survivorship was 75.8 % (IC95 % 63.3 %–84.5 %). In group R there was 4 cases of failures (33.3 %) of which 3 (25 %) for aseptic loosening; 40 moths cumulative survivorship was 63 %.

**Discussion** The two groups are numerically too much different for a comparison, but some conclusions can be made. Endo-Model prosthesis gave good clinical and radiographic results but, in consideration of the shorter survivorship compared to un-constrained prosthesis, its use is definitely reserved for cases of severe instability or revisions. The same considerations can apply to the model RHKnee but, concerning the failed cases, all the mobilizations occurred at the tibial level when the conical tibial stem was used. The use of cylindrical or longer stems is absolutely recommended.

**Conclusions** Both prosthetic models have demonstrated a cumulative survival lower than less constrained prosthesis. Therefore, their use for the first implants is restricted in cases of severe ligamentous instability.

### Endo-Model constrained knee prosthesis for first implant: 15-year follow-up

A. Bistolfi*[1], G. Massazza[2], F. Rosso[3], M. Paonessa[1], F. Galetto[1], A. Cimino[3], M. Crova[2]

[1]AO CTO M. Adelaide (Turin, IT);
[2]AO CTO M. Adelaide, Università degli Studi (Turin, IT);
[3]Università degli Studi (Turin, IT)

**Introduction** Knee constrained arthroplasties have been developed as an improvement of fixed hinge prosthesis. The purpose of this study is to evaluate clinical and radiographic results of a series of 98 implants with constrained prosthesis type Endo-Model® Link used in first plant.

**Materials and methods** From 1992 to 1995 98 Endo-Model were implanted in 84 patients (70 women), with an average age equal of 69.1 years (SD ± 7.4). Indications to surgery were: 67 knee arthritis due to axial defects (68.4 %), 21 Rheumatoid Arthritis (21.4 %), 5 sequelae of tibial plateau fractures (5.1 %) and 5 cases of knee arthritis consequent to OTAV (5.1 %). Average follow-up was 174.1 months. Hospital-Special-Surgery questionnaire was used for clinical evaluation and "Knee Society Roentgenographic Evaluation System" for radiographic ones. Data were collected in a prospectively; Log-rank or Wilcoxon test were used for statistical analysis and cumulative survivorship was calculated using Kaplan-Maier's method.

**Results** 61 patients (72 cases) entered the study. All HHS scores had a statistically significant increase. In particular, the range of motion increased from 88.2° (SD ± 7.6°) to 109.5° (SD ± 17.3°), and total score from 64.4 (SD ± 8.2) to 82.2 (SD ± 13.3). In 12 of 98 cases early complications occurred (12.3 %). In 9 cases progressive radiolucent lines were found; 7 of these failed. Cumulative survivorship was equal to 75.8 % (IC 95 % 63.3–84.5 %) at 15 years. Analyzing the survival of subgroups no statistically significant differences between implant with or without trochlea ($p = 0.30$) were noted, while a statistically significant difference was found between prosthesis with or without anti-dislocation system ($p = 0.03$).

**Discussion** Many authors suggest that these hinged prostheses are associated with increasing in stress transfer of bone-prosthesis or bone-cement interface, due to higher hinge. Nevertheless, rotating hinge prosthesis do not seem to have a highest risk of aseptic loosening due to the hinge. The cumulative survival of this series appears to be lower if compared to models with less constraint, but the hinge itself is not the first cause of failure.

**Conclusions** The use of this model in first prosthetic implants is indicated only in cases of severe instability, where it ensures good results against the complexity of surgical situations.

### Intra-operative quantification of patello-femoral joint kinematics in total knee arthroplasty and its correlation with femoral component position

A. Ensini*[1], C. Belvedere[2], A. Feliciangeli[1], A. Timoncini[1], V. Dedda[2], A. Leardini[2], S. Giannini[1]

[1]Clinica II, Istituto Ortopedico Rizzoli (Bologna, IT);
[2]Laboratorio Analisi del Movimento, Istituto Ortopedico Rizzoli (Bologna, IT)

**Introduction** In total knee arthroplasty (TKA) surgical failures can occur for patello-femoral joint disorders. Current knee surgical navigation systems provide femur/tibia tracking for relevant bone preparation and joint kinematics assessments, disregarding completely the patella in case of resurfacing. Patellar tracking is made difficult by the small dimensions of this bone and by TKA surgical maneuvres. Recently, a new technique has been developed for tracking the patella during TKA, which includes new technical/surgical instrumentation. The purpose of this study was to report on the first in vivo experiences of the intra-operative evaluation of tibio-femoral and patello-femoral joint kinematics in patients during TKA with patellar resurfacing. Correlation between patellar motion and femoral component position was also investigated.

**Materials and methods** Ten patients affected by primary gonarthrosis were implanted with a posterior-stabilized prosthesis with patellar resurfacing. All TKA were performed using a standard knee navigation system equipped with a specially-designed patellar tracker fixed onto the patellar anterior aspect. Femoral/tibial bone preparation and component implantation were performed according to standard navigated procedures. The patella was resurfaced and relevant resection plane was acquired by an instrumented probe. Final position of implanted components and lower limb alignment were also recorded. Tibio-femoral and patello-femoral kinematics were deduced from the anatomical survey, now including also patellar anatomical landmarks, and according to established recommendations and original proposals.

**Results** Patellar tracking was performed successfully in all TKA without complications in addition to standard evaluations, resulting in a maximum of 30 min longer operations. After TKA with patellar resurfacing, patello-femoral kinematics showed a mean (± standard deviation over the patients) range of flexion, tilt and medio-lateral shift respectively of 66.9° ± 8.5° (mean of minimum-of maximum, 15.6°–82.5°), 8.0° ± 3.1° (−5.3°–2.8°), and 5.3 ± 2.0 mm

(−5.5–0.2 mm). Significant correlations were found between the internal/external rotation of the femoral component and the range of patello-femoral tilt ($p = 0.05$; R = 0.64), and between the mechanical axis alignment on the sagittal plane and the range of patello-femoral flexion–extension ($p = 0.05$; R = 0.66) and antero-posterior shift ($p = 0.04$; R = 0.67).
**Discussion** This preliminary experience supports the relevance, feasibility and efficacy of patellar tracking in navigated TKA. These results reveal that patellar-dedicated measurements are of good value in patellar resurfacing. In non-resurfacing, a more proper femoral component positioning that takes into account also patellar motion can be also performed.
**Conclusions** These encouraging in vivo data may lay ground for the inclusion of procedures for patellar tracking in navigated TKA surgery for a more comprehensive assessment of the original knee and during all relevant surgical actions.

### Unicompartmental knee arthroplasty versus total knee arthroplasty in the same patient: a comparative study on 12 patients

V. Sessa[1], U. Celentano[1], U. Trovato*[2]

[1]UOC Ortopedia e Traumatologia, Ospedale Fatebenefratelli S. Giovanni Calibita (Rome, IT);
[2]Università Campus Biomedico (Rome, IT)

**Introduction** Unicompartmental knee arthroplasty (UKA) and total knee arthroplasty (TKA) are an option for surgical intervention for the treatment of single-compartment osteoarthritis. Few studies have compared UKAs and TKAs in the same patient. The purpose of this study was to compare the results of these two arthroplasties in a group of patients who had undergone both procedures, one on each knee.
**Materials and methods** We identified 10 patients (6 women, 4 men) with osteoarthritis who had a TKA on one side and a UKA on the other. Average patient age at the time of TKA was 77 years (range, 75–81 years), and at the time of UKA was 79 years (range, 73–83 years). Average body mass index (BMI) for the entire cohort was 29.17 (range, 23–33). The mean follow-up was 71 months (range, 51–100 months) and 46 months (range, 15–81 months) for TKA and UKA, respectively. Patients were evaluated using the Knee Society Score (KSS) and Knee injury and Osteoarthritis Outcome score (KOSS) (pain scores, function scores, range of motion (ROM), and radiographic data (including alignment measurements) were collected).
**Results** Preoperatively KSS averaged 64 points in both groups. At hospital discharge, the scores averaged 80 for UKA and 80 points for TKA. The KOSS scores were 68 UKA and 64 TKA, preoperatively and 82 UKA and 83 TKA at hospital discharge. At the latest follow-up, the overall mean KSS for UKA and TKA was 88 points and 87 points, respectively. The mean KOOS was 86 for UKA and 84 for TKA. There were no radiographic failures. Survivorship of the unicompartmental group was 85 % compared with 100 % in the total knee group.
**Discussion** Selection of appropriate patients and good surgical techniques are the key to getting good results in both surgical techniques. Unicompartmental knee is a good treatment option for patients with unicompartmental osteoarthritis especially for the minimally invasive technique for rapid recovery, good pain relief and excellent function that are suitable for the lifestyle of the patient.
**Conclusions** For knee function and postoperative pain, UKA appeared similar to TKA at 3 years follow-up. Range of motion was better in UKA compared with TKA. Complication rates after UKA and TKA appeared similar.

### Clinical pathway in gonarthrosis treated with TKR: the experience of an identical and integrated surgery-rehabilitation course

M. Franci*, P. Sommazzi, G. Cecchi, C. Tschallener, R. Mesolella, L. Pinzuti, A. Cappellari, D. Di Sabatino, S. Failli

Casa di Cura Frate Sole (Figline Valdarno, Florence, IT)

**Introduction** 142 patients suffering from gonarthrosis, 56 men, 86 women of the average age of 74.8 years old, operated by the same team with a standard surgery procedure using the same prosthetic model (Zimmer Nexgen LPS), they have taken an identical surgery-rehabilitation course from the first orthopaedic consultation up until a 12 month follow-up. The average admission has been for 18 days. The orthopaedic and rehabilitation wards adjoining and the same medical team has operated in a collective way. The physiotherapy treatment started in the orthopaedic ward, from the first post-surgery has continued in the rehabilitation department for 4 h a day up until the discharge. The team has carried out the analysis of the interdisciplinary management efficiency.
**Materials and methods** the cases are headed by following the same clinic management: (1) orthopaedic, clinic and radio-graphic evaluation by knee-score; (2) pre-surgery assessment addressed to the risk evaluation with settled surgery timing, security's parameters; (3) anticipating programming of specific strategies; global interdisciplinary's take charge at the time of the admission; (4) evaluation by FIM at the time of admission and discharge; (5) unique instrument's usage (PRI Piano Rehabilitative Individual) with specific attention to the motives that pushed the patient to surgery and his future expectations; (6) usage of a specific protocol for the sharing and standardization of the course; (7) evaluations on the modifiability of functional aspect shared with the patient; (8) determination of the aims/results of the assistance and the rehabilitation project; (9) a 12-month follow-up with clinical and radio-graphic evaluation by knee-score.
**Results** The functional information at discharge highlights a clear recovery of the autonomy (earned FIM admission/discharge 31.4). the results at 12 months confirm the recuperation for pain, ROM, stability, articular alignment (KS 27.5 ? 94.1),as in functional ambit (FS 47.1 $\geq$ 90.6). Statistical analysis shows a significant increase. 2 patients (1.4 %) have faced complications (TVS, acute pancreatitis). All patients have expressed a positive judgment on the efficiency of course and management of every step.
**Discussion** implementation of the course has brought a improvement in the work results, a major involvement of the patients and of the operators, a monitoring of the standards of quality and of the guide lines and a continuous improvement of the quality performance.
**Conclusions** the results obtained show the efficiency of the course in terms of rapid functional recovery and of consequence of minor social charge.

### *C10—SPINE 1*

### Percutaneous pedicle screw fixation in polytrauma patients

L. Proietti*, R. Maviglia, L. Scaramuzzo, G.R. Schirò, S. Sessa[1], E. Piervincenzi, V. Raggi, S. Cicconi, C. Fabbriciani

[1]Università Cattolica del Sacro Cuore (Rome, IT)

**Introduction** Aim of the study was to evaluate clinical results in polytrauma patients underwent to thoracic and lumbar open and percutaneous pedicle screw fixation on emergency versus delayed treatment.

**Materials and methods** The orthopaedic and anaesthesiologist clinical conditions of 24 patients, hospitalized from January 2009 to September 2011 were evaluated in a retrospective way. Patients were divided in 3 groups: (A) patients treated in a delayed time; (B) patients underwent to percutaneous pedicle screw fixation; (C) patients underwent to open pedicle screw fixation and fusion within 72 h. Patients were evaluated in term of age, accident dynamic, mechanical ventilation need, blood transfusion need, SAPS II, Apache II, Sofa score, last out-come and type of vertebral injury. To assure statistic homogeneity only patients affected by thoracic and lumbar amyelic fractures were evaluated.

**Results** No differences were seen in ICU stay between group B and C, longer ICU stay are registered for group A. In all groups a frequent association was seen with cranial and thoracic trauma. Mean SAPS II value was 30 (11–48) at ICU admission in group A, with a predictive percentage of mortality of 10.6 %, of 39 (9–61) in group B with a percentage of 22.9 % and of 24 (8–47) in group C with a percentage of 5.9 %. The mean Apache II score value was of 17 (5–31) in group A, of 13 (9–27) in group B, of 9 (7–13) in group C. No differences in SOFA score were observed in the 3 groups. Mean mechanical ventilation time was similar in group A 7.3 days (range 0–18) and B 7.3 days (range 0–20), longer mechanical ventilation time was observed in group C 8.3 days (range 0–18). Blood transfusion volume was lower in group B 300 ml (range 0–1,200 ml) versus group A 400 ml (range 0–1,000 ml) and group C 717 ml (0–3,000 ml). Only 1 patient in the delayed group died 8 days after ICU admission.

**Discussion** In polytrauma patients an early spine fixation improves the last clinical outcome. Patients underwent to percutaneous screw fixation showed a better outcome compared to the other two groups obtained despite worst clinical conditions and a higher predictive percentage of mortality.

**Conclusions** An early surgical treatment allows, also, in amyliec fractures, an easier nursing and a faster demission from ICU.

### *C11—TRAUMATOLOGY 3*

### Targeting of distal screws in tibial intramedullary nailing: comparison between the "free hand" technique and the SureShot® system

O. Moreschini*, L. Marcellini, R. Cannata, C. Villani

Sapienza Università di Roma (Rome, IT)

**Introduction** In the common surgery practice the intramedullary nail distal locking screws are inserted with the "freehand" technique. This procedure has always been quite successful but presents some issues: it can be technically demanding, time consuming, and expose the patient and medical staff to a considerable amount of ionizing radiation. In order to overcome these problems and improve the distal locking, a new technique has been developed. It allows the video guided targeting of the distal screws through the use of an electromagnetic field. The purpose of our study is to compare this new method with the conventional "freehand" technique.

**Materials and methods** 40 patients with tibial shaft fractures were treated by the same surgeon with intramedullary nailing. In 20 patients (group I) the distal locking was performed using the Trigen SureShot® (Smith & Nephew) system; in 20 patients (group II) the distal locking was performed with the "freehand" technique using the Trigen® (Smith & Nephew) nail. For each surgery the following parameters were considered: distal locking time and radiation exposure time.

**Results** The average of the distal locking time was 16.25 min for the group I and 22.55 min for the group II. the average of radiation exposure time during distal locking was 5.6 s. in the group I and 39.58 s. in the group II. This difference is statistically significant.

**Discussion** The comparison between the two methods shows that the Sureshot® system allows a reduction of time in distal locking but the most evident benefit is the reduction of the use of the fluoroscopy and the resulting decrease in the amount of radiation absorbed by medical staff and patient.

**Conclusions** The results are very encouraging because they show a significant decrease in the time of exposure to ionizing radiation, a shortening of the distal locking time and a very fast learning curve that makes this technique easier to be used by surgeons with less experience.

### Forearm acquired deformities

M. Massobrio*, G. Pellicanò, G. Corsi, P. Albanese, G. Antonietti

[1]Dip. Scienze App. Locomotore (Rome, IT)

**Introduction** Acquired and congenital pathologies can affect the forearm with severe deformities. Many studies have been conducted about congenital deformities treatment, but a classification for acquired deformities is missing. We conducted a clinical-radiographic study on a group of patients to classify these deformities and its results.

**Materials and methods** 13 cases of forearm deformities following traumas or performed treatment (from 2000 to 2011, 11 men and 2 women). Mean age 31 years (20–75 years). In 5 cases the initial treatment was incruent, in 8 cases it was surgical. In 7 patients 1 segment was affected (in 4 patients the radius, in 3 the ulna), in 6 patients both segments. Location assessment: 2 projections x-rays (AP and LL), including wrist and elbow. Deformity location: proximal, diaphisary, distal, defined with the abbreviation, in distal sense, R1, R2, R3 for the radius, and U1, U2, U3 for the ulna. We distinguish between the primary and secondary deformities, occurred later in a different location than the primary one. In wrist and elbow deformities, we assessed the valgus varus deformity, and also for the diaphisary deformities, varus and valgus angulation. The radial deformity was evaluated following Schemitsch and Richards classification, the functional results following Anderson classification. Six patients were operated with plate and screws, 6 cases with external fixation. One case was treated with bone resection. In 10 cases iliac crest bone graft, in 1 patient vascularized fibula.

**Results** In 13 patients with acquired deformity, in 4 cases the primary deformity, that affected the radial diaphysis (R2), determined a secondary deformity, in 3 cases in the distal ulna (U3) with a ulnar carpal dislocation, in 1 case in the distal radius (R3). Osteosynthesis treatment: in 1 case excellent result, in 5 satisfying. External fixation: in 1 patient excellent result, in 5 satisfying. Bone resection: satisfying.

**Discussion** Surgical treatments with osteosynthesis are the major causes of acquired deformities in adults. Location and etiology of the deformities are essential for the surgical indication and the result. It is important the restore the length of the deformed segment realigning the anatomical axis. X-rays allow distinguishing between primary and secondary forearm deformities.

**Conclusions** Characteristics and locations of post-traumatic deformities were identified. The major location is diaphisary and distal, the elbow is rarely affected. The functional consequence is a limitation in the hand range of motion. The best results are achieved with a short term treatment (within 3 months).

### *C12—SHOULDER AND ELBOW 3*

### Real-time sonoelastography of the rotator cuff

C. Tudisco*, S. Bisicchia, M. Stefanini, M. Antonicoli

Università di Roma Tor Vergata (Rome, IT)

**Introduction** Several variables are thought to influence the outcome, but the quality of muscles and tendons seems to be the most important. Sonoelastography (SE) is a non invasive method that uses ultrasounds to evaluate the mechanical properties of tissues (stiffness and elasticity), reflecting their quality. Real-time sonoelastography (RTSE) allows this features to be measured and displayed in real-time, as a color scale, thus being a non invasive, safe, cheap and reproducible device, potentially applicable in the clinical setting.
**Materials and methods** Fifty patients (32 male, 18 female) were prospectively enrolled in the study. All the patients were evaluated clinically with Constant and Murley score (CMS), Simple Shoulder Test (SST), Quick DASH and VAS for pain. Subsequently a RTSE of both shoulders was performed to determine the quality of the tendons. RTSE shows tissues with different elasticity in different colours, from red (soft) to blue (hard). RTSE was graded as grade $1 \leq 25$ % red colouring, grade $2 = 25$–$50$ % red colouring, grade $3 = 50$–$75$ % red colouring, and grade $4 \geq 75$ % red colouring.
**Results** The mean age of the patients was 62 (±5 years), the dominant arm was involved in 36 patients. The mean CMS was 61 (±5) points, the mean SST was 7 (±7) points, the mean Quick DASH was 3 (±1) points, the mean VAS for pain was 8 (±1). RTSE showed 15 grades 1 tendon, 24 grades 2, 8 grades 3, and 3 grades 4. For all statistical tests, the alpha level was set at 0.05. Statistical analyses were performed with SPSS v.15.0 (SPSS Inc., an IBM Company, Chicago, IL, USA).
**Discussion** There was a statistically significant association between clinical outcome and the grade of tendon quality at RTSE ($p < 0.05$).
**Conclusions** The quality of the rotator cuff tendons, evaluated by RTSE, strictly correlates with the clinical results of the patients.

### Outcome of single tendon rotator cuff repair in patients older than 65 years

A. Cozzolino*[1], A. Djahangiri[2] N. Helmy[2], M. Zanetti[2], C. Gerber[2]

[1]Villa dei fiori (Naples, IT);
[2]Uniklinik Balgrist (Zurich, Switzerland)

**Introduction** Age is considered a negative prognostic factor affecting rotator cuff repair outcomes and surgical reconstruction in elder people is debated. Aim of this study is to evaluate clinical and structural outcomes of rotator cuff repair in people older than 65 years.
**Materials and methods** 58 patients older than 65 years with reparable supraspinatus tendon tears underwent primary open (22 patients) or arthroscopic (36 patients) repair. Preoperative clinical evaluation, Constant Score (CS), X-rays and arthro-MRI were collected. A retrospective follow-up examination included a CS, X-rays and ecography.
**Results** 44 shoulders of 41 patients were followed up at a minimum of 24 months (mean 57, max 112). Mean age at operation was 70 years (66–80). In 31 cases (70 %) ecography showed complete tendon healing. In 3 open repair (18 %) and in 5 arthroscopic repair (19 %) was observed a re-rupture. CS improved from 49 pts (5–74) to 78 pts (23–100) ($p < 0.05$). Forward flexion improved from 128° (40°–180°) to 160° (90–180°) ($p < 0.05$). CS was better in people with intact rotator cuff tendon (82 pts; min 57–max 100) than in people with re-rupture (61 pts; min 23–max 88). 5 patients were reoperated for persistent pain or repair failure.
**Discussion** This study showed high efficacy of single sovraspinatus repair in people older than 65 years, which is in contrast with previous study. Such a difference can be explained with the inclusion criteria of this study. Probably an older age is associated to confounding factors like massive tear and muscular fat atrophy, weather in our study we included just people with small lesions and light muscular infiltration.
**Conclusions** Isolated sovraspinatus tendon repair in people older than 65 years has an high healing potential and shows good clinical and structural outcome.

### Surgical treatment of distal triceps tendon ruptures

G. Giannicola[1], F. Sacchetti*[1], G. Bullitta[1], M. Scacchi[1], M. Iapicca[2], G. Restuccia[3]

[1]"Sapienza" Università di Roma (Rome, IT);
[2]Ospedali Riuniti di Bergamo (Bergamo, IT);
[3]Azienda Ospedaliera-Universitaria Pisana (Pisa, IT)

**Introduction** Distal triceps ruptures represent less than 1 % of all tendon ruptures. They are frequently underestimated and, if not promptly and adequately treated, they can hesitate in a severe loss of upper limb function. Objectives of our study are 1) to analyze clinical results of tendon reattachment; 2) to perform histological examination of lesion edges 3) to evaluate clinical utility of post-operative MRI and isokinetic evaluation.
**Materials and methods** From 2008 to 2011, we treated 7 men mean aged 38 years. All tendon lesions were classified with MRI and surgical direct visualization. 4 out of 7 distal triceps ruptures were inveterate; there were 6 subcutaneous lesions and 1 stab wound. 6 patients were treated by transosseous reinsertion using Krackow type suture and 4 week immobilization. In the remaining patient, tendon reattachment was performed using suture anchors. In 6 out of 7 patients, triceps scar tissue was histologically analysed. Functional evaluation was assessed with MEPS, m-ASES and Q-Dash. Muscle strength was clinically evaluated at all follow-ups and using isokinetic evaluation at 12 months follow-up. Post-operative MRI was performed at 6 and 12 months in all patients.
**Results** 5 patients had a superficial rupture of long and lateral head of triceps tendon, while 2 patients had a superficial and deep rupture. Histological examination revealed degenerative alterations in 5 of the 6 subcutaneous lesions; they were associated with dystrophic calcification in 33 % of cases. At the last follow-up, all patients had excellent results in term of ROM and functional evaluation. Manual test showed triceps strength to be 5/5 in all cases; isokinetic peak strength was 80 % of the uninvolved extremity, while the isokinetic work was 90 %. There were two complications: 1 deep infection with partial retear of tendon and 1 suture wire intolerance with wound problem.
**Discussion** Surgical treatment of acute and chronic triceps tendon lesions allows achieving excellent results. An important risk factor for subcutaneous tendon lesion is degenerative alterations. MRI is essential for diagnosis and classification of lesions and, together with isokinetics, it is useful to follow the healing process and to plan rehabilitation programs and the return to daily and sport activities. Isokinetic evaluation showed a satisfactory but incomplete strength recovery.
**Conclusions** Surgical treatment of triceps tendon lesions allows obtaining satisfactory clinical results in most of cases. Late diagnosis determines a greater complexity of surgery but it does not seem to affect the final clinical outcome.

### Treatment options of humeral shaft nonunion

G. Campochiaro*, S. Bondioli, L. Malagoli, M. Gialdini, C. Rovesta, F. Catani

Policlinico di Modena (Modena, IT)

**Introduction** The incidence of nonunion of humeral shaft due to delays of consolidation and infectious processes can reach values up to 10 %. The methods for the treatment of this disease may be different and the results are variable. We reviewed a series of cases treated in our center with different surgical alternatives and sought the most appropriate solutions for this condition.
**Materials and methods** From 2003 to 2011 we treated 23 patients with nonunion of humeral shaft. These patients were previously treated with different surgical techniques (9 with intramedullary nail, 8 with plates and screws, 5 with external fixation, 1 with metal cerclage). All patients had a bone gap less than 5 cm. 7 patients were treated with intramedullary nail and bone graft, whereas 16 patients with plate and screws plus bone graft (autologous and/or homologous). The mean follow-up was 16 months (min 6–max 60). Patients were clinically evaluated by DASH score and Constant score and radiographically.
**Results** We obtained healing in 85 % of cases. Concerning the complications, 3 patients had broken synthesis and they needed a new operation, 1 patient had a radial nerve lesion and 1 infection.
**Discussion** As reported in the literature, the most frequent cause of nonunion of humeral shaft is the inadequacy of initial surgical treatment, in particular the use of devices that do not provide axial and rotational stability. The quality of the stumps, the possible loss of bone substance and the conditions of the soft tissues have a fundamental role in the choice of nonunion treatment. A sensitive and important time in prediction of functional recovery is the management of radial nerve.
**Conclusions** The treatment of humeral shaft nonunion is a tricky challenge for the orthopaedic surgeon. The methods of treatment are different and the results are variable. In patients with bone gap less than 5 cm, we proved better results using plates plus bone graft and platelet gel, in accordance with the international literature.

### Long term quality of life in patients treated for massive retracted immobile cuff tears

P. Randelli, P.A. Arrigoni, C. Fossati*, L. Zottarelli, A. D'anna, P. Cabitza

Policlinico San Donato, Università degli Studi di Milano (San Donato Milanese, Milan, IT)

**Introduction** The aim of this study is to evaluate long term quality of life outcomes of arthroscopic repair in patients with massive contracted immobile rotator cuff tears.
**Materials and methods** We included 25 patients (26 shoulders) in this study with massive contracted rotator cuff tears, either partially or completely, arthroscopically repaired between 2005 and 2009. In 18 cases (70 %) the cuff was completely repaired to the bone using an interval slide technique (single or double interval slide in 11 and 7 patients respectively). In the remaining 8 shoulders (30 %) a functional partial rotator cuff repair was performed (in 3 of them the repair was performed using an interval slide technique). Patients were retrospectively evaluated with validated outcomes scores: Disabilities of the Arm, Shoulder and Hand questionnaire (DASH), Visual Analog Scale (VAS) for pain, Simple Shoulder Test (SST) and Single Assessment Numeric Evaluation (SANE). Range of motion (ROM) was determined by a self-assessment questionnaire.
**Results** The mean age was 64.1 (range 49–74). The mean post-operative follow-up was 39.6 months (range 19–70). A overall satisfactory quality of life was reported (76 % mean SANE). The final mean DASH and SST was respectively 23.8 and 8.8. The residual level of pain was low (mean VAS 1.8). Mean range of motion were 157.5° in elevation and 55.3° in extra-rotation.
**Conclusions** Arthroscopic repair in massive contracted, immobile, rotator cuff tears provide a long term satisfactory quality of life. This minimal invasive surgical approach can be a valid alternative to reverse shoulder arthroplasty.

### *C13—KNEE 3*

### Comparative analysis of international scores as outcome measures in gonarthrosis

G. Cerulli*[1], I. Illuminati[2], F. Fantasia[3], G. Placella[4], E. Sebastiani[4], P. Antinolfi[6], G. Potalivo[5]

[1]Nicola's Foundation Onlus, Let People Move Research Institute (Arezzo, IT);
[2]Università degli Studi di Perugia (Perugia, IT);
[3]Dipartimento di Ortopedia, Nuova Clinica San Francesco (Foggia, IT);
[4]Clinica Ortopedica e Traumatologica, Università degli Studi di Perugia (Perugia, IT);
[5]Nicola's Foundation Onlus, Let People Move Research Institute, International Orthopedic and Traumatology Institute (Arezzo, IT);
[6]Clinica Ortopedica e Traumatologica, Ospedale Santa Maria della Miseicordia (Perugia, IT)

**Introduction** Quality of Life (QoL) is a personal subjective perception which can be measured by self-administered questionnaires. Several health-related QoL instruments are available but their validity, reliability and responsiveness must be carefully evaluated before they should be used.
**Materials and methods** Forty-five patients with gonarthrosis undergoing total knee arthroplasty were enrolled and randomly assigned to three different groups, fifteen patients each. The scores we evaluated are: WOMAC, SF-36, KOOS, IKDC and LYSHOLM. These are the most frequently disease-specific and generic scores used in gonarthrosis. The aim of the study was to compare the sensitivity of pain grading and physical function for each score.
**Results** The spearman's correlation for validity was statistically significant between subscale ADL (KOOS) and Pain (SF-36) with a coefficient of 0.5 and between Function in sport and recreation (KOOS) and Role-Physical (SF-36) with a coefficient of 0.5. Lysholm showed a strong correlation with the subscale Symptoms (KOOS) with a coefficient of 0.7. Reliability was evaluated using Cronbach's alpha where values greater than 0.8 are considered acceptable. The Alpha value lies between 0.36 and 0.89 for KOOS's five subscales, between 0.84 and 0.90 for WOMAC's subscales; lower values were observed for IKDC (alpha = 0.47). To assess responsiveness Standardised Response Mean and Effect Size were evaluated. Disease-specific scores (four out of five) have demonstrated a high responsiveness (=0.8) and SF-36 have a moderate-high responsiveness.
**Discussion** To analyze data we performed parametric and nonparametric procedures because the scales and subscales of the different scores have a non-Gaussian distribution.
**Conclusions** The subscales that measure physical function showed a good validity, and we feel that they represent an adequate measurement system also in terms of patients' understanding of the questions. We found a high grade of internal consistency (=0.60) for Pain, Function in daily living, Function in sport and recreation and Quality of life (KOOS) and for all WOMAC's subscales (=0.84). IKDC has a lower responsiveness than KOOS and WOMAC, but in general the disease-specific scores showed a higher responsiveness than SF-36.

### Long-term, perspective comparison of FB versus MB TKA

A. Bistolfi*[1], M. Paonessa[1], F. Rosso[2], D. Deledda[2], F. Galetto[1], G. Massazza[2], M. Crova[2]

[1]AO CTO M. Adelaide (Turin, IT);
[2]Università degli Studi (Turin, IT)

**Introduction** Total knee arthroplasties have demonstrated good long term clinical results. Fixed tibial plateau models have shown implant's survivorship between 90 and 98 % at 10–15 years of follow-up. Mobile tibial surface knee arthroplasties have been designed in order to improve the clinical outcomes and survivorship of the implant. However no studies have demonstrated the superiority of mobile surfaces on fixed ones. Aim of this study is to compare long term results of Nexgen® prosthesis both with mobile and fixed surface.
**Materials and methods** Between 1998–2002 in a continue series of patients with the same clinical characteristics 100 knee arthroplasties with fixed plate and 100 with mobile one were implanted. This prosthesis were implanted in 163 patients, 136 women and 27 men; average age was 70 years (range 43–86). Surgery technique was the same in all patients. All clinical and radiological data were analyzed with a statistical significance level equal to $p = 0.05$.
**Results** Average follow-up was 116 months. At follow-up ending 8 patients (10 knees) died, no one of which has been implant's revision. 18 patients (18 knees) were lost to the last follow-up. Average KSS-KS score at the last control was 85.2 (IQR 82.89: min. 58.8, max. 98) points for fixed plate group and 86.8 (IQR 83–90; min. 71, max. 98) points for mobile surface one ($p = 0.06$). Radiolucent lines (2 mm) were found in 12 knees (7 fixed plates and 5 mobile surface). In 2 knees (1 fixed plate and 1 mobile one) progressive radiolucent lines were found, and the implants was revised. 3 fixed plate prostheses (1 aseptic loosening and 2 bilateral instabilities in the same patient) and 1 mobile surface were revised.
**Discussion** The two models have shown good clinical, radiographic and survival results at 10 years without statistically significance differences in term of clinical results and survivorship between the two groups ($p = 0.33$). Actually, indication to use mobile surface arthroplasty is based on theoretical possibilities to obtain best long term clinical results, in absence, at this moment, of results that confirm this choice.
**Conclusions** There is no evidence of better results in implants with fixed plate *versus* mobile ones and contrary.

### Treatment of ostechondral lesions of the knee with Maioregen® bio-mimetic scaffold (Finceramica Faenza S.p.A, Italy): 2-year results

M. Berruto, G. Carimati*, E. Usellini, F. Uboldi, G. Vergottini, L. Gala

Istituto Ortopedico G. Pini (Milan, IT)

**Introduction** Ostechondral lesions of the knee are very common disorders in young adult population. Due to associated symptoms, limited healing capacity and possible development of secondary osteoarthritis an effective treatment is mandatory.
**Materials and methods** From December 2008 to October 2011 we treated 17 patients (14 men, 3 women). Average age was 42 years (min 15, max 47). Patients presented osteochondritis dissecans disease (acute or chronic) in 9 cases, osteonecrosis in 2 cases and focal degenerative osteochondral lesions (excluding diffuse osteoarthritis) in 6 cases. Patients were evaluated by subjective and objective IKDC score, Lysholm Knee score and Visual Analogic Scale (VAS) before surgery and at 6 and 12 months follow up. Only 9 patients were evaluated also at 24 months follow-up. All cases underwent MRI before surgery and at follow ups, which were evaluated with MOCART score.
**Results** At 18.3-month average follow-up all patients presented a mean value of IKDC subjective scale of 73.41 and 84.82 on the Lysholm Knee score. Medium VAS score was 2.4. At 1 year follow up the mean IKDC subjective score was 69 (56.65 before surgery) and the mean Lysholm score was 86.35 (63.65 before surgery). The medium increase at 1 year was 26 % on the Lysholm scale and 18 % on the subjective IKDC scale. We observed on the 9 patients who were able to be evaluated at 24 months a medium increase of 24 % on the Lysholm scale comparing to before surgery but the score was 2 % lower comparing to 1 year follow-up. The medium increase at 24 months follow up on the IKDC subjective scale was 21 % comparing to before surgery and 11 % comparing to 1 year follow up. Medium VAS score were 5.58 before surgery, 3.6 at 1 year follow up and 1.4 at 24 months for only 9 patients. At 1-year follow-up mean VAS value decreased of 2.52 points while in the 9 patients evaluated at 2 years mean VAS value decreased of 4.18 points.
**Discussion** Maioregen is a three-dimensional multi-layer matrix which mimics the entire osteocartilagineous tissue and is able to induce a different differentiation of cells into osteocytes in deepest part and chondrocytes in the joint surface in order to obtain a better reparation of the osteochondral defect.
**Conclusions** Maioregen represents an effective treatment for recent osteochondral lesions ICRS grade 3 and 4 of the knee with limited cost, easy surgical technique and promising results at 1 year minimum follow-up.

### Comparative evaluation of the lateral notch sign in patients with anterior cruciate ligament tear

E. Paiusco*, M. Ronga, G. Gianazza, P. Cherubino

Dipartimento di Ortopedia e Traumatologia, Università dell'Insubria (Varese, IT)

**Introduction** The lateral notch sign (LNS) is a depression of the lateral femoral condyle, visible on X-rays in lateral view, determined by the impact of the condyle with the region of the upper postero-lateral tibial plateau during knee sprains that cause ACL tear. Aim of this study is to perform a comparative evaluation of LNS on X-rays of patients with ACL injury. The hypotheses of the study are: LNS is an indirect radiographic sign of ACL injury; there is no inter-and intra-individual variability in the evaluation of the LNS; the LNS is associated with a specific type of knee trauma; the LNS is associated with meniscal lesions.
**Materials and methods** 80 consecutive patients with ACL tear were included in the study. X-rays of both knees were performed before surgery. The LNS was measured by drawing the perpendicular to the tangent of the depression of the condyle. Nakauchi classification was adopted to evaluate the LNS type. The frequency and depth of the LNS was evaluated independently by two residents of the first year of orthopaedics course. Three evaluations were performed, at a distance of 1 week from each other, by one of the observers randomly chosen. The results were correlated with the type of mechanism of trauma, the presence of meniscal tears, the age, sex and the sport practiced by the patient at the time of trauma.

**Results** The frequency of the LNS was for single observer 36 of 80 patients (45 %) and 34 of 80 patients (42.5 %) respectively ($p > 0.05$). The average depth was 1.3 mm (range, 0.5–2.9 mm) and 1.2 mm (range, 0.5–2.9 mm) ($p > 0.05$). There were no significant differences in the classification of the LNS and the three different measurements of the individual operator. The LNS was associated mainly with valgus and external rotation trauma injury and to the lateral meniscal tear ($p < 0.05$). There were no correlations between LNS and other variables.

**Discussion** The LNS is a reliable and easily detectable indirect radiographic sign of ACL tear. The association of valgus and external rotation trauma with LNS and the positive clinic signs of lateral meniscus tear supports the hypothesis of ACL rupture.

**Conclusions** Future goal is to continue long-term study of the same patients to assess the possible clinical and radiographic degenerative changes of this lesion.

### Non-invasive assessment of Pivot-Shift test in ACL injury with triaxial accelerometer KiRA

F. Uboldi*[1], L. Gala[3], G. Vergottini[1], W. Albisetti[4], S. Zaffagnini[5], M. Berruto[6]

[1](Milan, IT);
[2](Bologna, IT);
[3]Scuola di Specialità di Ortopedia e Traumatologia, Università degli Studi di Milano (Milan, IT);
[4]Università degli Studi di Milano (Milan, IT);
[5]Istituto Ortopedico Rizzoli (Bologna, IT);
[6]Istituto Ortopedico G. Pini (Milan, IT)

**Introduction** Lachman test and Pivot-Shift test are the two most commonly used clinical tests to evaluate laxity of the anterior cruciate ligament (ACL) of the knee. The antero-posterior laxity can be measured in mm with some devices like KT1000® instrument; whereas there is not a validated tool on the market that can measure the rotational instability.

**Materials and methods** KiRA (Kinematic Rapid Assessment) is a device created and patented by the Biomechanics Laboratory of the Orthopaedic Institute of Bologna. KiRA is a triaxial accelerometer that is placed on the tibia, it allow us to measure the acceleration of the limb during the Pivot-Shift test. The obtained data are transferred to a pc by Bluetooth connection, then the software shows the acceleration curve of the tibia and an algorithm isolates the values of the test allowing comparison with the contra lateral limb. Fifty patients were evaluated with KiRA, they were aged between 16 and 45 years. The patients had a partial or complete lesion of the ACL, they were evaluated preoperatively or postoperatively; we performed also a standard Lachman test and Pivot-Shift test.

**Results** Preliminary results indicate a good correlation between the value measured by the tool and the assessment of the clinical tests performed by the surgeon. The results obtained comparing the values of the injured and the contralateral limb seam equally promising.

**Conclusions** At this early stage we observed a operator-dependency of the results, they seem to be much more reliable when detected by an experienced surgeon.

### *C14—SPINE 2*

### Correction of vertebral rotation with posterior arthrodesis in idiopathic scoliosis

F. Lolli*, M. Di Silvestre, T. Greggi, F. Vommaro, E. Maredi, A. Baioni, K. Martikos

Chirurgia delle Deformità del Rachide, Istituto Ortopedico Rizzoli (Bologna, IT)

**Introduction** There is still much debate upon the techniques of vertebral derotation during correction of idiopathic scoliosis by posterior approach. The objective of this study is to evaluate two different techniques of direct derotation.

**Materials and methods** We evaluated 42 consecutive patients affected by adolescent idiopathic scoliosis, treated surgically in our Division by posterior instrumented arthrodesis with pedicle screws. Inclusion criteria were: (1) adolescent idiopathic scoliosis Lenke type 1 or 2; (2) posterior instrumentation with pedicle screws; (3) direct vertebral rotation technique (DVR); (4) minimum follow-up of 2 and a half years. Axial rotation of apical vertebrae was evaluated with CT scan before and after operation (mean 7 days), by using Aaro and Dahlborn criteria.

**Results** All 42 patients were revisited. We considered 2 groups: in one group (22 cases) derotation was performed before the application of the two rods (Pre-rod) while in the other one (20 cases) it was performed after the rotation of the concave rod. We did not find any differences in terms of age, Risser sign, type of curve, Cobb angle, curve corrigibility, extension of arthrodesis and sagittal profile between the two groups. In the Pre-rod group we obtained a significantly better correction of vertebral rotation (Pre-R 61.4 % vs. Post-R 54.8 %; $p < 0.05$) and a better final correction of scoliosis (63.4 % vs. 61.1 %; ns). T5-T12 kyphosis, that was similar between the two groups before operation (Pre-R 16.9° vs. Post-R 17.5°), resulted instead inferior in the Pre-Rod group at final follow up, when compared to the Post-Rod group where kyphosis was incremented (Pre-R 12.5° vs. Post-R 18.5°). Instead lumbar lordosis, which was similar between the two groups before operation (−41° vs. −42.1°) resulted similar at final follow up (−44.9° vs. 43.2°). The questionnaires' scores were also similar at final follow up between the two groups (SRS-30 and SF-36).

**Discussion** The most efficient technique for correction of vertebral rotation (and also for correction of Cobb angle) was derotation performed before rod insertion (Pre-Rod). The reduction of kyphosis that was registered in the Pre-Rod group was avoided when derotation was performed after concave rod rotation (Post-Rod).

**Conclusions** Both techniques appeared effective. We obtained better results in terms of derotation with the Pre-Rod technique but this strategy implied a worse result in terms of sagittal aspect of the spine.

### Neuromuscular scoliosis: current surgical techniques

T. Greggi*, M. Di Silvestre, F. Lolli, A. Ortolani, E. Maredi, A. Baioni

Chirurgia delle Deformità del Rachide, Istituto Ortopedico Rizzoli (Bologna, IT)

**Introduction** In literature there are still controversies if posterior only fusion is a sufficient procedure in the treatment of neuromuscular scoliosis. Aim of our study is to evaluate long terms results of neuromuscular scoliosis treated at Our Department with posterior only instrumented fusion and to test the safety and the effectiveness of this technique.

**Materials and methods** From January 1995 to January 2009, 41 consecutive patients, surgically treated at our Department by posterior only instrumented fusion, were reviewed. There were 20 females and 21 males with a mean age of 15.8 years (range 10–38). Diagnosis was: cerebral palsy (13 cases), Duchenne muscular dystrophy (7), spinal amyotrophy (7), myelomeningocele (5), poliomyelitis (3), Friedreich's ataxia (2), Escobar syndrome (2), Steinert's disease (1), Charcot Marie Tooth disease (1). Main scoliosis Cobb angle averaged 94.05° (range 34°–165°), the curve was thoracic in 19 cases, thoracolumbar or lumbar in 22 cases. Kyphosis (T5-T12) averaged 42.86° (range 7°–90°), lordosis was 33.57°. The fusion was extended to the lumbar tract in 23 patients, to the sacrum in the other 18.

**Results** The mean follow-up was 11.8 years (range 10–14). Among 23 patients with fusion extended to the lumbar tract, scoliosis decreased from a preoperative value of 96.94° (range 65°–139°) to a postoperative value of 68.41° (range 20°–100°), with a mean correction of 30.33 % (range 6.6–69 %). Loss of correction at follow up was 4.13 %. Complications occurred in 6 patients, and revision surgery was performed in 5. Among 18 patients with fusion extended to the sacrum, scoliosis decreased from a preoperative value of 91.73° (range 34°–165°) to a postoperative value of 55.73° (range 8°–110°), with a mean correction of 38.43 % (range 0–76 %). Loss of correction at follow-up was 4.95 %. Complications occurred only in 2 cases, requiring in both cases a revision surgery.

**Discussion** In both groups, posterior fusion only permitted to obtain a significant correction of the scoliosis, stable at follow-up, with a low complications incidence.

**Conclusions** Our results showed that, in patients with neuromuscular scoliosis, posterior instrumented fusion is a safe and effective procedure and is the treatment of choice for patients with limited respiratory function, as in Duchenne muscular dystrophy and spinal muscular atrophy. The surgery should be performed as early as possible, and the extension of the fusion to the sacrum should be avoided in patients with residual walking ability.

### Algorithm for the treatment of penetrating spinal injury

L. Amendola*, M. Cappuccio, G. Bosco, L. Mirabile, S. Paderni, F. De Iure

Ospedale Maggiore (Bologna, IT)

**Introduction** The penetrating spinal traumas are extremely rare in European population and the most common form is caused by a firearm or weapon. In surviving patients, the severity of injury depends on the anatomical regions involved: spinal canal penetration almost always leads to neurological damage.

**Materials and methods** Five patients were identified in our hospital as having sustained penetrating spinal injuries from 2000 to 2012. Upon arrival at the emergency room, all of them showed stable vital signs, so they were subjected to clinical and radiological examination. In one case the knife was removed without wound exploration as neuro-vascular structures were not involved. In a penetrating unstable vertebral fracture with Brown-Sequard syndrome caused by hemisection of the spinal cord, surgical exploration was indicated to remove the blade safely and to performed posterior stabilization. In one patient with incomplete neurologic deficit the awl had been removed by the same assailant so an MRI was performed after CT scan to assess the cord injury: he had a complete neurologic recovery without surgery. In a T8 fracture with a complete neurological deficit the bullets have been removed and resection and diverting ileostomy was performed cause by small bowel associated lesion. An extremely comminuted and unstable gunshot fracture of L1 was treated by decompressive laminectomy and posterior fusion.

**Results** A complete neurologic recovery occurred in only one case. No patient experienced complications and were discharged or transferred to other structure after a mean of 5 days.

**Discussion** The management of penetrating spinal injury includes: (1) CT scan enhanced with contrast is the first level examination to define the trajectory/position of the weapon respect to the spinal cord and other vital structures and to assess the degree of bone lesion; (2) MRI is performed whenever metal foreign bodies are excluded by CT-scan and in case of neurologic symptoms to define the cord injury and possible hematoma in the spinal canal; (3) surgery is performed to remove persistent metal object in the spinal canal or in the vertebral column.

**Conclusions** A diagnostic and operative protocol based on our experience and review of the literature is presented.

### Posterior fixation in the treatment of post-traumatic cervico-thoracic instability

G. Bosco*, M. Cappuccio, S. Paderni, G.B. Scimeca, L. Mirabile, L. Amendola, F. De Iure

UOSD Chirurgia Vertebrale, Ospedale Maggiore (Bologna, IT)

**Introduction** Trauma of the cervico-thoracic junction with instability, is an infrequent condition. The diagnosis and treatment of these lesions are an important issue for spinal surgeons. The aim of this paper is to review our experience in the treatment of traumatic injuries of cervico-thoracic junction with posterior instrumented arthrodesis.

**Materials and methods** Between January 2006 and December 2010, 32 patients with an unstable post traumatic lesion in C6-T3, underwent to posterior fixation in our Institution. There were 28 males and 4 females, mean age 53 years (min. 20–max. 84). Sixteen patients had neurological deficits at presentation. In all cases stabilization was obtained by implantation of pedicle screws in the thoracic tract and in C7 and screws in the articular masses in other cervical vertebrae, connected with titanium rods. In no patients were used hooks. In 11 cases an anterior approach was performed. In all cases with cord involvement was associated urgent decompression. The mean follow-up was 18 months (min. 6–max. 58). The sagittal and frontal alignment and screw placement were evaluated with postoperative imaging. All patients were periodically followed in outpatient clinic.

**Results** There were no reported failures of mechanical implants, and no patient was subjected to further revision surgery. In all cases there has been the complete bone fusion within 6 months after surgery. In no case was a worsening of neurological conditions and in 60 % of patients with cord involvement was observed partial or complete improvement of neurologic status.

**Discussion** The cervico-thoracic junction is a complex anatomical region, the lesions in this area are a significant clinical problem both in terms of correct diagnosis that of choosing the most appropriate surgical treatment. The positioning of pedicle screws in C7 and in the first thoracic vertebrae, while requiring adequate learning curve, allows excellent results regarding the stability and the correction, with a low incidence of complications.

**Conclusions** In our experience, limited to cases assessed, the posterior arthrodesis has proven an effective technique in the treatment of lesions of the cervico-thoracic junction.

### *C15—TRAUMATOLOGY 4*

### Stability plate surgery in proximal humerus fractures: deltoid-pectoral approach versus direct lateral transdeltoid approach.

S. Cigni*[1], E. Battista[2], A. Chessa[1], L. Pietrogrande[3]

[1]AO San Paolo, Polo Universitario (Milan, IT);
[2]Facoltà di Medicina e Chirurgia, Università degli Studi di Milano (Milan, IT);
[3]UO Ortopedia e Traumatologia, AO San Paolo, Polo Universitario, Facoltà di Medicina e Chirurgia, Università degli Studi di Milano (Milan, IT)

**Introduction** Proximal humerus fracture incidence is increasing. Classification and treatment are not definitely codified still. The aim of this study is to compare evidences after angular stability plate surgery through deltoid-pectoral approach versus direct lateral transdeltoid one.
**Materials and methods** 40 patients (12 males and 28 females) underwent surgery for proximal humeral fracture employing an angular stability plate. The patients were divided in two groups: group A underwent deltoid-pectoral approach, group B direct lateral. Mean age was 64 year olds. All patients were evaluated with clinical examination, X-rays and Constant, Quick Dash, UCLA and Oxford score.
**Results** In both groups right side and females were the most involved. Mean operative time was 120 min. After surgery a shoulder support was maintained for 25 days. Assisted rehabilitation had a mean starting time of 35 days and went on for 3 months. No bone necrosis was detected on X-rays. A partial humeral head collapse was seen in one case. We had a temporary disestethic syndrome in group A and a circumflex nerve paresis in group B. Skin complications were seen in 2 cases in group B. Articular shoulder stiffness was detected in 4 cases in group A and one case in group B. Direct lateral approach was faster, and allowed a better elevation and internal rotation recovery; stiffness was lower and a better subjective satisfaction was obtained. All scores showed better results: Constant, Oxford, UCLA and Quick Dash. All patients had better subjective functional recovery in daily activities. Deltoid-pectoral approach gave better cosmesis and a better abduction and extrarotation recovery.
**Discussion** Surgery in proximal humeral fractures has a generally high rate of complications. Surgeon target becomes an anatomical reduction with a good stabilizing surgical technique and a fast surgery time. Patients' compliance and daily life goals have always to be considered. The patient must obtain a good pain control and perform a fast rehabilitation to prevent stiffness, ROM limitation and force reduction. Postoperative immobilization must be reduced to minimal.
**Conclusions** Patients with proximal humerus fractures requires an accurate evaluation concerning indication to surgery. Surgeon must take care of surgical technique. Assisted rehabilitation has to be started as soon as possible; shoulder immobilization must be maintained for few days. Direct lateral approach in our series gave better functional results but deltoid-pectoral was better for abduction recovery. Deltoid muscle required accuracy during rehabilitation. Remaining clinical and instrumental evidences were similar in both groups.

### Vancouver classification: lights and shadows

S. Pelle*[1], A. Formica[2], A. Mattei[2], L. Magistro[2], A. Impagliazzo[3]

[1]Università degli Studi di Roma "Sapienza" (Rome, IT);
[2]Azienda Ospedaliera San Giovanni Addolorata (Rome, IT);
[3]Casa di Cura Addominale EUR (Rome, IT)

**Introduction** Vancouver classification proposed by Duncan and Masri on 1995 became the most widely accepted classification system of periprosthetic femoral fractures around hip prostheses. Recent studies confirmed reliability and validity of this classification system. (Rayan F, Dodd M, Haddad FS (2008) European validation of the Vancouver classification of periprosthetic proximal femoral fractures. J Bone Joint Surg Br 90-B (12) :1576–1579). Therefore, while recognizing its merits, several authors (Jiranek W, 2011; Park S et al., 2011;. Ninan TM et al., 2007; Lindahl H et al., 2010; Hou Z et al., 2011) remarked its important pitfalls.
**Materials and methods** Through a review of our cases, we found the reliability of this classification, but we also noticed some imperfections that, if not carefully interpreted and evaluated by the surgeon, could induce wrong therapeutic indications.
**Discussion** On one hand, the classification of Duncan and Masriproposes a practical algorithm able to provide reproducible useful therapeutic indications based primarily on three simple parameters (lights): 4. location of the fracture; 5. Femoral stem stability; 6. periprosthetic bone quality; on the other hand, its validity is undermined by some considerations (shadows); 7. "timing" is not evaluated; 8. it does not provide criteria to define the stability of the prosthesis stem (subtypes B1 and B2 are often confused); 9. the assessment of bone quality and of fixation is conducted only for the group B, and not for the A and C subtypes; 10. it does not consider acetabular component and periacetabular bone; 11. Possible infections are not contemplated; 12. it does not consider age and functional status of the patient as well as it does not evaluate the time elapsed since the first prosthesis.
**Conclusions** We therefore hold that Vancouver classification in clinical practice should not be regarded as an absolute guide to the type of treatment, but therapeutic indications must be interpreted case by case basis and require a special experience by the surgeon.

### The fractures of the tibial plateau: treatment algorithm

P. Barbato*, W. Leonardi

ARNAS Garibaldi "Nesima" (Catania, IT)

**Introduction** The fractures of the tibial plateau represent an important understood it in traumatology, to high energy express a high damage to the structures and the function of the articulation of the knee. To the anatomical member who for the biomechanics which this district is subordinate, needs of an adapted surgical treatment that you respect to the maximum its natural physiology. Therefore it demands one high and real surgical experience apt to reconstruct the anatomy of the articulation in order to allow an optimal resumption of the articular heads.
**Materials and methods** From 1999 to 2011 we dealt with 283 patients, 67 % were subjected to surgical treatment with an age ranging between 18 and 70 years with a peak ranging between 30 and 50 years. We used Shatzker classification. In some fractures of 3° and all of the 4°, 5° and 6° type we used the hybrid external fixator. These allowed us dealing with the bony damage for its necessary reconstruction without furtherly damage woven soft that cover the articulation and the leg. Often a lot of damages from extended phlyctenule contraindicate the surgical participation.
**Results** For the clinical evaluation we used the table of Hohl-luck, modified from JENSEN et al. (1990), for the radiographic evaluation we used the criterion of Bernischke (1992). In the circular assemblies we had 70 % of good and 15 % excellent results, as concerning the hybrid assemblies 69 % were bonds, 18 % excellent. No case of osteomyelitis.
**Discussion** The use of this technique allowed us dealing with the fractures reducing the damages to the soft woven, guaranteeing premature use of the knee. In average the patients had the full load of the limb at 45 days. The clinical recovery was in average at 150 days.

**Conclusions** Our aims were the anatomical reduction, the stable synthesis and the premature recovery of the functionality. The external fixation is a valid technique of synthesis for the attainment of these aims. The recovery worked considerably well and it was easier compared with that of the traditional surgery.

### *C16—SHOULDER AND ELBOW 4*

### Clinical and radiographic results of acute acromioclavicular type III dislocations: low incidence of scapular dyskinesis and sick scapula syndrome at long term follow-up

L. Murena, G. Canton*, E. Vulcano, M. Surace, P. Cherubino

Dipartimento di Biotecnologie e Scienze della Vita, Università degli Studi dell'Insubria (Varese, IT)

**Introduction** The ideal treatment for acute type III acromioclavicular (AC) dislocations is controversial. In fact, the literature reports little evidence on which treatment (surgical versus conservative) results in better clinical outcome. Recently, evidences for long term better outcome of surgical treatment have been published in literature, and development of scapular dyskinesis and SICK scapular syndrome following conservative treatment was described.
**Materials and methods** We evaluated 34 patients surgically treated for acute type III AC dislocations with different techniques. Functional outcome, radiographic evaluation of clavicular reduction and scapular kinematics were all evaluated.
**Results** Excellent Constant shoulder score (mean 95.7 points, SD ± 5.3) and Simple Shoulder Test (mean 11.2 points, SD ± 0.8) results were observed. In 4 patients recurrence of separation was observed. Scapular dyskinesis was observed in only 4 (11.7 %) patients, of which only 1 (2.9 % of the sample) was affected by SICK scapula syndrome. Scapular dyskinesis was classified as type I in 3 cases (75 %) and type III in 1 case (25 %).
**Discussion** Excellent Constant shoulder score (mean 95.7 points, SD ± 5.3) and Simple Shoulder Test (mean 11.2 points, SD ± 0.8) results were observed. In 4 patients recurrence of separation was detected. The incidence of scapular dyskinesis and SICK scapula syndrome in the present study was significantly lower with respect to conservative treatment. On the other hand, the distribution of dyskinesis types was comparable, suggesting a possible common cause. However, pathogenesis of scapular dyskinesis following type III AC dislocation is still not clear: periscapular muscle dynamic stabilisation seems to be the means by which the scapula correctly accomplishes its functions, while the results hereby presented do not allow surely establishing a correlation between clavicle reduction and scapular dysfunctional syndromes.
**Conclusions** Excellent clinical results for surgical treatment of type III acute AC joint dislocations at long term follow up are confirmed. Surgical treatment is also associated with a lower incidence of scapular dyskinesis and SICK scapula syndrome if compared to conservative treatment.

### The complex instability of the elbow: treatment strategy

R. Tucci*, R. Angeloni

(Florence, IT)

**Introduction** Elbow dislocation can produce ligaments and capsule lesions so to determined instability joint. These lesions associated an elbow fractures (radial head, coronoid fractures or both) create a severe instability joint, the so called "the complex instability of the elbow" This report is showing the outcomes of the treatment proposed by Pugh et al. [1].
**Materials and methods** This retrospective study examines twenty patients treated in our Institution, Shoulder and Elbow Unit of the CTO Florence Hospital, between 2005 and 2011. The people were affected by fractures-dislocation of the radial head in ten cases, fractures-dislocation of the coronoid in six cases and dislocation with both fractures (the so called "terrible triad") in 4 cases. We recorded data using MEPS (mayo elbow performance score). We implanted radial head prosthesis in three patients because were affected by severe radial head fractures. In nine people a dynamic external fixator was used because at the end of the procedure (treatment of the bone and the ligaments injuries) the elbow had showed persistent instability.
**Results** At average follow-up 28.2 months (6–72) we have recorded data of seventeen patients and the results were excellent 41 % (7 patients), good 47 % (8 patients), sufficient 3 % (one patient) and poor 3 % (one patient). The average flexion movement was 118.8°, while the average extension motion was 14.2°. The people treated with dynamic external fixator have had average ROM 108.7°, while people treated without device had average ROM 98.1 %. None of the elbows had symptoms or sign of instability at final evaluation.
**Discussion** The treatment of the elbow complex instability is very difficult and the main target is to restoring a elbow stable, painless and functional. A dynamic external fixator can be used to stabilize the joint during an optimal movement.
**Conclusions** In the complex instability of the elbow the principles osteosynthesis are fundamental to obtain a good functional outcome and a stable elbow. In cases where this principles aren't sufficient the main priority is to repair the ligament lesions and finally to apply the dynamic external fixator so to favour early elbow motion during healing ligaments.

**Reference**

1. Pugh DM, Wild LM, Schemitsch EH, King GJ, McKee MD (2004) Standard surgical protocol to treat elbow dislocations with radial head and coronoid fractures. J Bone Joint Surg Am 86:1122–1130.

### Radial head arthroplasty: mid-term results

F. Mosetto*[1], S. Vanni[2], A. Martinasso[2], G.P.V. Vasario[3], B. Battiston[3], P. Tos[4]

[1]UOC Ortopedia, Presidio Sanitario Gradenigo (Turin, IT);
[2]Prima Clinica Ortopedica, Università di Torino (Turin, IT);
[3]UOC Traumatologia Muscoloscheletrica, ASO CTO M. Adelaide (Turin, IT);
[4]UOD Microchirurgia, ASO CTO M. Adelaide (Turin, IT)

**Introduction** The radial head is a secondary stabilizer of the elbow. Prosthetic implant to restore the stability of the elbow is indicated in case of fractures that cannot be synthesized or sequelae of synthesis and resections in complex lesions. The purposes of this study were to review the medium term results obtained with the use of radial head prosthesis.
**Materials and methods** 20 patients undergone radial head arthroplasty were reviewed at a 25 months follow-up (minimum 12). 17 patients were treated early: 7 patients had Mason 3 type fracture, 10 patients had a Mason 4 type fracture. In 12 cases they showed bone or ligament associated injuries that were treated as follows: fractures of the coronoid with reconstruction by anchors or synthesis, lesions of the medial or lateral collateral ligaments with suture anchors or through direct suture, fractures of the olecranon with plates and screws, residual instability with positioning of articulated external elbow fixator. 3 patients were treated for bad outcome of previous treatments (2 surgical and 1 nonsurgical) and underwent joint replacement, arthrolysis, re-creation of capsuloligamentous complex in 1 case and ulnar neurolysis in 1 case. All the patients were subjected to clinical evaluation to investigate the overall functionality, through medical examination and several questionnaires (VAS, DASH, MEPS, Liverpool Elbow Score-LES, Oxford Elbow Score) and to radiographic evaluation for the detection of general complications of elbow trauma (such as arthritis, ossification) and specific complications related to prosthetic implant (such as asymmetry of the joint line, areas of osteolysis, loosening or breakage signs).
**Results** The average total clinical and functional results were as follows: flexion 126°, extension deficit 20.5°, pronation 67.6°, supination 59.5°, VAS 2.4, DASH 17.3, MEPS 80.5, Oxford Elbow Score 36.8, SLE 7.6. By means the radiographic evaluation we found: 9 cases of osteoarthritis, 13 cases of ossifications, 6 of asymmetry of the joint line, 3 of periprosthetic osteolysis, no sign of loosening or breakage of the prosthetic implant. In 3 cases it was necessary a second operation in 1 case for rigidity, in 1 case for implant revision and in another one for removal of the means of synthesis from the ulna. Patients resulted satisfied in 85 % of the cases.
**Conclusions** The radial head arthroplasty has proven to be an effective solution for the early treatment of radial head fractures and for late management of adverse outcomes with previous treatments. These results reflect those found in the literature.

### Percutaneous pinning of 3- or 4-part fractures of proximal humerus in elderly patients in poor general conditions: MIROS® versus traditional pinning

S. Carbone*[1], M. Tangari[2], S. Gumina[1], R. Postacchini[3], A. Campi[2], F. Postacchini[1]

[1]Ortopedia e Traumatologia, Università di Roma Sapienza (Rome, IT);
[2]Ortopedia e Traumatologia, Ospedale San Giovanni-Addolorata (Rome, IT);
[3]Ortopedia, Ospedale Israelitico (Rome, IT)

**Introduction** Elderly subjects often have fractures of the proximal humerus, which may be difficult to manage in patients in poor general conditions. The MIROS is a new percutaneous pinning device allowing correction of angular displacement and stable fixation of fracture fragments. We evaluated the results of percutaneous fixation of 3- or 4-part fractures of proximal humerus of patients in ASA (American Society of Anesthesiologists) physical status 3 or 4 treated either with MIROS or traditional percutaneous pinning (TPP).
**Materials and methods** 31 patients treated with MIROS and 27 undergoing TPP were enrolled in the study. Preoperatively, anteroposterior and transthoracic or axillary radiographs were obtained in all cases and CT scans in patients with most complex fractures. Follow-up evaluations were carried out at 3, 6, 12 and 16 weeks, and 6 months, 1 and 2 years postoperatively, using the Constant Score (CS) and Subjective Shoulder Value (SSV) methods.
**Results** 52 out of the 58 patients could be evaluated at all follow-ups. In both 3- or 4-part fractures there were significantly higher CS and SSV scores in the MIROS compared to TPP group at all late follow-ups. A lower rate of deep infection, pin tract infection and pin mobilization were found in the MIROS group ($p < 0.001$). In both groups there was a significant association between final result (CS) and either the type of fracture and complications ($p < 0.001$).
**Discussion** Treatment of complex proximal humerus fracture is difficult, in particular in elderly subjects who often have precarious general conditions. In addition, there is no evidence in literature that open reduction and internal fixation is better than closed reduction and external fixation in all ages.
**Conclusions** The MIROS gave better clinical results and less complications than TPP in elderly patients. This method, however, may not be indicated for younger patients in good general conditions.

### Treatment of simple elbow dislocation with deformable plastic cast and immediate mobilization against resistance

A. Poggetti*[1], D. Ciclamini[1], B. Panero[1], P. Titolo[1], R. Matteotti[2], B. Battiston[2], P. Tos[2]

[1]I Clinica Ortopedica Universitaria (Pisa, IT);
[2]Dip. Ortopedia e Traumatologia, UOD Microchirurgia, ASO CTO M. Adelaide (Turin, IT)

**Introduction** Elbow dislocations are classified as simple or complex types. The simple dislocation is characterized by the absence of fractures, but if not correctly treated and rehabilitated may present complications. The aim is to describe the modern treatment of simple elbow dislocation using deformable thermoplastic plastic cast and immediate mobilization against resistance of the splint.
**Materials and methods** From 2009 to 2011 at the UOC of Traumatology of the CTO in Turin were treated 14 patients for simple elbow dislocation. The patients used a brace brachio-metacarpal in Dynacast® (immediate active mobilization into range of stability) maintained full-time for 21 weeks then removed and maintained only night. The patient is asked to move actively against the resistance of the brace in flexion and extension.
**Results** The follow-up was clinical and radiographic. There were no major complications. An average deficit of extension of 10° was present, the flexion was not limited. The pronation was reduced of 20° in 5 cases and supination of 30° in 9. There was a direct relationship between contracture and age of the patients. No recurrence of dislocation appeared and an easy rehabilitation program started at the end of the treatment without pain and co-contraction of muscle.
**Discussion** The simple elbow dislocations can be treated with close reduction and early mobilization. The use of "cheap and easy" plastic cast that permits movements against resistance increases compliance and limits the long-term complications leading a more" functional" healing without losing proprioception.
**Conclusions** The deformable plastic cast represent an excellent choice of treatment of simple elbow dislocations that allow an immediate mobilization with an early start of rehabilitation program and a quick return to daily activities.

### *C17—KNEE 4*

### Retrospective clinical and radiographic study on 50 cases of bilateral knee prosthesis

S. Giannotti*, V. Bottai, A. Gentile, G. Dell'Osso, G. De Paola, N. Cazzella, G. Bugelli, G. Guido

Clinica Ortopedica, Università di Pisa (Pisa, IT)

**Introduction** This paper aims to analyze, through special evaluation systems, the clinical and radiographic results obtained in patients who underwent bilateral knee replacement performed in 2 steps. We also evaluated the psychosomatic profile of patients and the presence of comorbidity factors to highlight a possible relationship/influence between these and the clinical outcome.

**Materials and methods** Between 2003 and 2010, the Orthopaedic Clinic at the University of Pisa, about 50 patients underwent bilateral knee replacement. We performed a retrospective clinical and radiographic review able to determine the clinical outcome with a follow-up period of about 52.5 months from first treatment and approximately 30.7 months from the second. The patients were evaluated by a "clinical objective" and a "subjective" point of view, using score evaluation: Knee Society Score (KSS), Knee Society Function Score, Oxford Knee Score (OKS) and WOMAC. Radiologically, using the Knee Society score, it was evaluated the prosthetic alignment and the presence of radiolucent lines.
**Results** We had no cases of replanting except the 2 cases of patellar resurfacing occurred at a later time. The "clinical score" made for each operated limb ranged between a minimum value of 20 and a max of 100, the average was 86.5. The "Functional Score" global per patient ranged from a value of 20 min and a max of 100, the average was 77. This indicated that the overall clinical outcome was excellent and the functional one was good.
**Discussion** In our experience we show that patients with comorbidity factors, such as anxious-depressive syndromes, obesity, CVI, despite an objective clinical and radiographic good results, had pain in at least one of the two knees with a consequent decline of joint function. We have seen the contrast between the medium good results on KSS and poor functional outcomes at KSS Function Score, OKS and WOMAC scores.
**Conclusions** Considering that our study evaluated 40 implants for a total of 80 bilateral implants and that in the literature no one work was focused on a sample consisting exclusively by bilateral implants, we can say that our results are placed in the foreground.

### MPFL reconstruction in objective patellar instability. Results comparison between autologous and biosynthetic tendon with an original surgical technique

G. Vergottini*[1], F. Uboldi[1], E. Usellini[2], L. Gala[3], M. Berruto[2]

[1](Milan, IT);
[2]I.O. G. Pini (Milan, IT);
[3]Scuola di Specialità di Ortopedia e Traumatologia, Università degli Studi di Milano (Milan, IT)

**Introduction** In the last decade, the role of the medial patellofemoral ligament (MPFL) as the primary stabilizer of the patella, has been increasingly emphasized. Patellar dislocation or subluxation can cause a lesion of the MPFL in more over 90 % of cases. The treatment of chronic medial patellofemoral ligament tears is now considered one of the most useful surgical techniques to stabilize the patella.
**Materials and methods** In this study we analyzed 19 patients with objective patellar instability (9 males, 10 females, mean age 21.5 years, range 17–32) who underwent, from 2007 to 2011 (follow-up 1–5 years, mean 2.5 years) surgical reconstruction of the MPFL with the original technique (two tunnels transrotulei) using biosynthetic LARS tendon (12 cases) or autologous ST (5 cases) or allograft (2 cases). Among the analyzed 19 patients, 4 cases underwent a reconstruction of the MPFL without surgical peripheral association. We associated the medialization of the ATA in 7 cases and the lowering of the ATA in 2 cases. In the remaining 6 cases, we performed both procedures (medialization and lowering). The patients were evaluated clinically and functionally at a mean follow-up of 29 months (range: 12–60 months) with subjective scales (Koos score and Kujala score) to assess satisfaction and with clinical examination to test the patellar stability and tracking. After analyzing radiographs and CT scans, we evaluated the height of patellar tilt and analyzed any pathological patellar tilt and patellar tunnels condition.
**Results** From a subjective point of view, patients expressed an excellent opinion in 15 cases and good in the remaining 4. We did not record any case of instability recurrence or failure of the ligament. One case only had to be surgically re-operated because of the presence of scar adhesions on femoral medial epicondyle (on femoral tunnel). In 90 % of the cases we reported normalization of the pathologic parameters evaluated pre-operatively. From a radiological point of view we found the incomplete ossification of the bone tunnel at a distance, without substantial differences in the use of autologous tendon or synthetic one.
**Discussion** The reconstruction of the MPFL patellar provides stability and improves the clinical symptoms.
**Conclusions** According to "Lyonnaise school" we believe that to achieve good results the MPFL reconstruction can be associated to other procedures to correct any pathological factors leading to instability.

### Partial anterior cruciate ligament tears: anatomical reconstruction technique versus non-anatomical augmentation technique

R. Buda*, A. Ruffilli, F. Tesei, G. Pagliazzi, A. Parma, M. Baldassarri, M. Bulzamini, S. Giannini

Istituti Ortopedici Rizzoli, II Clinica (Bologna, IT)

**Introduction** Partial anterior cruciate ligament (ACL) tears occur in 28–35 % of knee sprains with hemarthrosis. The preservation of the intact ACL bundle brings several advantages, including increased mechanical stability in the post-operative period, an afferent vascular compliance and preservation of the proprioceptive system. Aim of this study was to compare the clinical results of an anatomical reconstruction technique (AR) of the ACL injured bundle, compared to a non-anatomical augmentation technique (NAA) with femoral over-the-top fixation (OTT).
**Materials and methods** Fifty-two patients (mean age 23.3 years) with partial ACL tears underwent an operation: 26 patients received a AR, 23 a NAA. Two patients were found intra-operatively to have a complete lesion, requiring a traditional reconstruction of the ACL. Patients were evaluated by IKDC scale, Tegner score and arthrometer KT-1000, pre-operatively, at predefined step and at the final follow-up of 5 years.
**Results** At the final follow-up IKDC subjective was 88.2 ± 5.7 in the AR group and 90.2 ± 4.7 in the NAA group. IKDC objective showed 96 % of normal knees in the NAA group and 87.5 % in the AR group. The reconstruction of the antero-medial bundle (AM) showed subjective and objective results significantly worse than those obtained from the reconstruction of the postero-lateral bundle (PL) ($p = 0.017$).
**Discussion** Surgical treatment of partial ACL tears is a technically demanding procedure. Appropriate portals, careful reaming of the tunnel and an intercondylar space control are key points for a successful AR technique. Although there is no statistically significant difference in terms of objective and subjective results between the two techniques, we found an higher incidence of instability in mid-long term with AR technique.
**Conclusions** The NAA technique is a less technically demanding procedure with better results and fewer complications. The reconstruction of the AM bundle provided poorer results, especially if performed with AR technique.

### *C18—SPINE 3*

### Posterior lumbosacral interbody fusion with the Bartolozzi screw

M. Di Silvestre*[1], T. Greggi[1], F. Lolli[1], A. Baioni[1], P. Bartolozzi[2]

[1]Chirurgia delle Deformità del Rachide, Istituto Ortopedico Rizzoli (Bologna, IT);
[2]Clinica Ortopedica e Traumatologica (Verona, IT)

**Introduction** Lumbosacral interbody fusion is necessary in the surgery of severe spondylolisthesis, of III and IV grade: it can be obtained with different techniques, such as PLIF or TLIF by posterior approach with incremented duration of surgical procedure and blood loss, or by combined anterior approach (ALIF). The objective of our study is to evaluate the results of the Bartolozzi screw used to perform interbody fusion in the posterior surgical treatment of severe spondylolisthesis.
**Materials and methods** We evaluated 11 cases, with a minimum follow up of 12 months, affected by severe spondylolisthesis of L5, aged between 15 and 45 years: patients received posterior pedicle screw fixation and also posterior stabilization with a Bartolozzi screw from the sacrum to the vertebral body of L5 (titanium cylindrical screwed cage, with a diameter of 7 mm, filled with autologous bone graft). In these cases affected by III or IV grade spondylolisthesis, reduction was only partially obtained; since it was not possible to perform an interbody fixation with PLIF or TLIF because of the technical difficulties related to residual listhesis, and unwilling to perform an anterior complementary approach, we introduced a Bartolozzi screw from the sacrum for interbody fusion L5-S1. Procedure was rapid and easy (mean 15 min).
**Results** We did not have any neurologic complications and non mechanical complications were registered at follow-up, with stability of listhesis partial reduction obtained during operation.
**Discussion** The Bartolozzi screw permitted to obtain an interbody fusion with a posterior only approach.
**Conclusions** Interbody fixation with the Bartolozzi screw represents an efficient and secure procedure in respect to other interbody fusion techniques, by offering an immediate "also anterior" stability in highly dysplastic incomplete listhesis reduction, performed by an all-posterior approach.

### Bilateral transpedicular facet augmentation Percudyn system for the treatment of lumbar spinal stenosis

R. Iundusi*[1], S. Masala[2], P. Pistillo[1], V. Tempesta[1], E. Gasbarra[1], G. Simonetti[2], U. Tarantino[1]

[1]U.O.C. Ortopedia e Traumatologia B, Fondazione "Policlinico Tor Vergata", Università degli Studi di Roma "Tor Vergata" (Rome, IT);
[2]Dipartimento di Diagnostica per Immagini, Fondazione "Policlinico Tor Vergata", Università degli Studi di Roma "Tor Vergata" (Rome, IT)

**Introduction** Lumbar spinal stenosis is a collection of pathologic degenerative changes involving all the anatomical structures that compose the classic "three-column" complex. Spinal stenosis prevalence has been calculated to range between 3.9 and 14 % among patients with low back pain.
**Materials and methods** From January 2010 to October 2011 twenty-four consecutive patients suffering spinal stenosis signs and symptoms, resistant to conservative treatment, severe enough to let them eligible to posterior laminectomy-foraminotomy were proposed to a new minimally invasive interventional alternative treatment by implant of a new posterior stabilization device Percudyn SystemTM Anchor and Stabilizer (Interventional Spine Inc., Irvine, CA) under local anaesthesia and fluoroscopic guidance. All Patients underwent preoperatively lumbar flexed-extended X-ray to rule out spinal instability, CT and MRI to assess the type and degree of stenosis. MRI has also been performed in upright position to rule out dynamic stenosis worsening. Follow-up clinical examinations have been scheduled at months 1, 6 and 12 after procedure. Outcome has been evaluated by SF-36 Bodily Pain (SF-BP) and Physical Function (SF-PF) 0–100 scales in which higher scores indicate less severe symptoms, Oswestry Disability Index (ODI) 0–100 scale in which lower scores indicate less severe symptoms, Stenosis Frequency and Bothersomeness Index (SFI and SBI) 0–24 scales in which lower scores indicate less severe symptoms, Low Back Pain and Leg Pain Bothersomeness Index (LBPBI and LPBI) 0–6 scales in which lower scores indicate less severe symptoms.
**Results** Twenty patients (83 %) progressively improved during the one-year follow-up. Four (17 %) patients did not show any improvement and opted for surgical posterior decompression. In no case, both responder and not-responder patients, any device related complication was reported. We were not able to detect any significant difference in both clinicalelectromyographic setting and imaging findings being their characteristics completely superimposable to those of the responding patients. A finding common to all these four patients was the resistance of their symptoms to forward bending.
**Discussion** Percudyn System is a pedicle screw based posterior stabilization device which allows load transfer to the articular facets meantime preventing spine hyperextension, whose rationale is to avoid facet joint overlapping by blocking them in a slightly flexed position so gaining enough foraminal and spinal channel room and leading to clinical improvement.
**Conclusions** Minimally invasive PDS Percudyn System has shown effective and safe in treating selected cases of lumbar stenosis patients.
